# Inference of a Boolean Network From Causal Logic Implications

**DOI:** 10.3389/fgene.2022.836856

**Published:** 2022-06-16

**Authors:** Parul Maheshwari, Sarah M. Assmann, Reka Albert

**Affiliations:** ^1^ Department of Physics, Penn State University, University Park, PA, United States; ^2^ Biology Department, Penn State University, University Park, PA, United States

**Keywords:** Boolean network inference, Boolean model, network inference, network construction, stomatal closure, guard cell

## Abstract

Biological systems contain a large number of molecules that have diverse interactions. A fruitful path to understanding these systems is to represent them with interaction networks, and then describe flow processes in the network with a dynamic model. Boolean modeling, the simplest discrete dynamic modeling framework for biological networks, has proven its value in recapitulating experimental results and making predictions. A first step and major roadblock to the widespread use of Boolean networks in biology is the laborious network inference and construction process. Here we present a streamlined network inference method that combines the discovery of a parsimonious network structure and the identification of Boolean functions that determine the dynamics of the system. This inference method is based on a causal logic analysis method that associates a logic type (sufficient or necessary) to node-pair relationships (whether promoting or inhibitory). We use the causal logic framework to assimilate indirect information obtained from perturbation experiments and infer relationships that have not yet been documented experimentally. We apply this inference method to a well-studied process of hormone signaling in plants, the signaling underlying abscisic acid (ABA)—induced stomatal closure. Applying the causal logic inference method significantly reduces the manual work typically required for network and Boolean model construction. The inferred model agrees with the manually curated model. We also test this method by re-inferring a network representing epithelial to mesenchymal transition based on a subset of the information that was initially used to construct the model. We find that the inference method performs well for various likely scenarios of inference input information. We conclude that our method is an effective approach toward inference of biological networks and can become an efficient step in the iterative process between experiments and computations.

## 1 Introduction

Network inference from expression information is an information extraction process where the inputs are knowledge of the identity of the components that make up a network and their states in a variety of contexts, and the output is a proposed regulatory network with edges and functions that define the dynamics between the biomolecules. For inference of a gene regulatory network, the input information comes from gene expression data, e.g., RNA-seq assays. Signal transduction networks can be inferred from data on protein expression and post-translational modifications, combined with information on small molecule mediators. Metabolic networks may be inferred from the knowledge of metabolite and enzyme concentrations. Various methods have been developed for network inference; each of these methods have their strengths and weaknesses.

Correlation measures (e.g., Pearson correlation coefficient) of the expression of gene or protein pairs can be used to construct a weighted gene or protein co-expression network (Huang et al., 2005). The correlation measures can also be combined with clustering methods such as hierarchical clustering or K-means clustering to obtain groups of co-expressed genes/proteins (Horvath et al., 2006). These networks show the extent of co-expression between genes/proteins and may not be indicative of whether the gene products/proteins regulate each other or have any causal influence. Probabilistic graphical models like Bayesian networks use Bayesian inference to obtain conditional regulatory functions that indicate the probability that a target node has a certain state given the state of its regulators. This inference method often necessitates extensive data to calculate the conditional probability of the state of the target node given the state of the regulators (Sachs et al., 2005).

Network construction using edge inference from causal information (such as information from perturbation experiments) is a general method, applicable to any system, that represents an efficient alternative to network inference from state information (Albert et al., 2007; Kachalo et al., 2008, S.; Li et al., 2006). The input information is the identity of the components that make up a network and causal relationships between them, and the output is a proposed regulatory network. The causal effects used as input information include the positive or negative causal effect of one node on another (A → B), or information of the positive or negative effect of a node on the regulation of another node by a regulator (A → (B → C)). We will refer to the latter as a three-node causal effect. The inferred network incorporates each two-node causal effect as an edge or path of the corresponding sign. Experimentally documented direct interactions are always represented by edges. The inferred network incorporates each three-node causal effect as the intersection of two paths of the corresponding sign. Specifically, (A → (B → C)) will yield a positive path from B to C and a positive path from A to C, which intersect at an unknown mediator (a pseudo-vertex). Two reduction algorithms have been developed to simplify the resultant network while preserving each of the initially encoded causal relations: binary transitive reduction with critical edges, and pseudo-vertex collapse (Albert et al., 2007; Kachalo et al., 2008). The resulting network is the most parsimonious incorporation of the input information. This network synthesis method has been applied to various biological systems and resulted in equivalent networks compared to manual curation (Kachalo et al., 2008).

The Boolean modeling framework has been used successfully to model the dynamics of various types of biological networks (Wynn et al., 2012; Saadatpour and Albert, 2013; Abou-Jaoudé et al., 2016) as well as for model inference from state (e.g., gene/protein expression or post-translational modification) data. Boolean models assume two possible states of each node, 1 (which can be interpreted as ON, active or above-threshold level) and 0 (interpreted as OFF, inactive, or below-threshold level). When a Boolean framework is used for network inference, a key pre-processing step is to discretize the data to either 0 or 1. Several methods are used for discretization of the relevant data for inference (Berestovsky and Nakhleh, 2013). One example is iterative k-means clustering where the data are iteratively clustered into fewer clusters until there are only two clusters that correspond to ON and OFF. The discretized data are then interpreted as Boolean states (e.g., activity). The inference process (described below) is performed in the same way independent of the entity whose state is described by the input data.

A traditional method to infer a regulatory network and Boolean functions from state information is to observe the time-course of the states of each node and perform an exhaustive search through all possible Boolean functions (with all subsets of nodes as possible regulators) to find the one that best fits the given data (Pandey et al., 2010; Berestovsky and Nakhleh, 2013; Dinh et al., 2017). This method is implemented in the software BoolNet (Müssel et al., 2010). This exhaustive search can be very time-consuming. Another difficulty is that it is often the case that not all of the combinations of the putative regulators’ states are observed experimentally; thus, the inference is under-constrained and can be satisfied by multiple alternate set of regulators and multiple functions for each particular node.

A more effective method is to combine prior network information with state data (for example, known attractors or trajectories of the system) to infer the Boolean functions. Several methods preserve the prior knowledge network during the process of inferring the Boolean functions (La Rota et al., 2011; Ghaffarizadeh et al., 2017; Chevalier et al., 2020; Aghamiri & Delaplace, 2021). Such methods are implemented in the applications Griffin and SMBionet (Khalis et al., 2009; Munoz et al., 2018). Other methods refine the starting network by deleting or adding edges (Terfve et al., 2012; Azpeitia et al., 2013; Abou-Jaoudé et al., 2016; Dorier et al., 2016). Iterative experimental and computational analysis can then be used to further refine the Boolean network.

Here, we present a combined network and Boolean function inference method based on causal logic relationships between different network components (inferred from perturbation experiments), extending the work in Albert et al. (2007), Kachalo et al. (2008). We utilize the abundance of genetic or pharmacological perturbation (knockout and overexpression) experiments in the biological literature to infer causal logic relationships. We then infer a parsimonious network and a set of Boolean functions that recapitulates these causal relationships. Our method differs from other Boolean network inference methods in that it does not require snapshots or time courses of all the nodes’ states, nor does it require a prior knowledge network. Our method covers the middle ground between curated (manual) network and model construction and automated network inference. It is closer to the former in that it aims to find the most parsimonious model and does not explicitly identify all the alternative models. Because of this reason, the resulting model should be verified by follow-up experiments, as all models should. This streamlined network and model inference method is aimed at making the model construction process less laborious and hence making it more accessible to the larger biological community.

## 2 Materials and Methods

### 2.1 Background: Causal Logic Implications Between a Pair of Nodes in a Boolean Network

Causal logic, introduced in (Maheshwari and Albert, 2017), identifies causal relationships between pairs of nodes in a Boolean network as sufficient or necessary. This logic implication tells whether the sustained activity of the regulator node is sufficient or necessary to activate the target node (for a promoting edge) or deactivate the target node (for an inhibiting edge) regardless of the state of other regulators.

There are four categories of logic relationships between a regulator and its direct target: sufficient activator, sufficient inhibitor, necessary activator, and necessary inhibitor. All of these relationships are independent of the state of any other regulators. In other words, these are canalizing relationships (Kauffman, 1993). The logic relationships are summarized in Table 1. In the following we give two examples. If the sustained ON state of a regulator node leads to the sustained ON state of the target node, we say that the regulator is sufficient for the target. A regulator node being necessary for a target node means that the sustained OFF state of the regulator node leads to the sustained OFF state of the target node. Such necessary relationships are abundant in biology; for example, in an enzyme-catalyzed reaction both the presence of the reactant(s) and the activity of the enzyme are necessary for the production of the reaction’s product.

**TABLE 1:** Summary of the six different types of causal logic implication and their correspondence with the direct effect of the state of the regulator node (R) on the state of the target node (T). The first column lists the causal logic implication, the second column lists what that implication indicates about the definite knowledge of the state of the target node if the state of the regulator node is known, and the third column lists the corresponding Boolean rules. The “…” in the Boolean rule is a placeholder for any number of other regulators of the target node. The asterisk (*) denotes a future state of a node, i.e., T* refers to the future (or next timestep) state of the target node.

Causal logic implication	What does it mean for the state of T (independent of the state of the rest of the network)?	Equivalent Boolean rule
Sufficient	R = ON => T = ON	T* = R or …
Sufficient inhibitory	R = ON => T = OFF	T* = not R and …
Necessary	R = OFF => T = OFF	T* = R and …
Necessary inhibitory	R = OFF => T = ON	T* = not R or …
Sufficient and necessary	R = OFF => T = OFF	T* = R
	R = ON => T = ON	
Sufficient and necessary inhibitory	R = OFF => T = ON	T* = not R
	R = ON => T = OFF	

An indirect regulator to target relationship can also have a logic implication; this relationship is mediated by a path or subgraph between the regulator and target node. For example, an indirect sufficient relationship between R and T can be mediated by a group of mediators M_i_ such that each M_i_ is necessary for the target node, the union of M_i_ is collectively sufficient for T, and R is sufficient for each Mi; see (Maheshwari and Albert, 2017) for a description of all the paths and subgraphs that mediate a logic implication. In these latter cases, the logic implication is independent of all other nodes in the network except for the nodes that make up the path/subgraph of the indirect regulation. An especially salient relationship is the combination of sufficient and necessary logic implication, i.e., when the state of a target node is completely determined by the state of a distant regulator node. A sufficient and necessary promoting relationship means that the state of the target node will be the same as the state of the regulator node while a sufficient and necessary inhibitory relationship means that the state of the target node will be the opposite of the state of the regulator node. More details on each of these causal logic relationships can be found in Maheshwari and Albert (2017).

### 2.2 Combining Causal Implications Incident on the Same Target Node

In a large and complex network, nodes can have multiple direct regulators, each of which may have a different causal logic implication on the target node. These logic implications must correspond to a single Boolean function that preserves each logic implication. Consequently, the resulting Boolean function is in the family of biologically meaningful functions (Raeymaekers, 2002) (i.e., no regulator is redundant or has an ambiguous effect), and also in the family of nested canalizing functions (Y. Li et al., 2013). Only certain combinations of logical regulators are able to preserve each logic implication. To see why this is the case, consider a hypothetical situation in which a target node (T) has a direct regulator (R1) that is sufficient. According to the definition of a sufficient regulator, the ON state of R1 always implies the ON state of T independent of the state of other regulators. In terms of Boolean functions, the existence of a sufficient direct regulator among multiple regulators implies a logic OR gate. This means that the effect of R1 is compatible with another direct regulator R2 that is also sufficient, making the update function T* = R1 or R2. Here T* indicates the next state of the target node T. The other case of compatibility is when R2 is a necessary inhibitor; in this case the function of the target is T* = R1 or not R2. Node T cannot have another direct regulator (R2) that is necessary, because the “necessary” classification of R2 (i.e., the OFF state of R2 implies the OFF state of T) contradicts the sufficiency of R1. In summary, sufficient regulators are incompatible with necessary regulators. Please note that this incompatibility does not mean that every regulator’s effect on the target must always combine with a logic OR relation. For example, the Boolean rule for a target node can be T* = R1 or (R2 and R3). Here, neither of the regulators R2 or R3 are independently sufficient or independently necessary for T but they are still compatible with regulator R1.

We summarize the compatible logic implications in Table 2 and describe them in words in the following. When a direct regulator is sufficient and necessary, it must be the only regulator of the target node. Similarly, when a direct regulator is sufficient and necessary inhibitory, it must be the only regulator of the target node. Necessary regulators are compatible with other necessary regulators and any other sufficient inhibitory regulators. Sufficient inhibitory regulators are compatible with other sufficient inhibitory regulators and any other necessary regulators. Sufficient regulators are compatible with other sufficient regulators and any necessary inhibitory regulators. Necessary inhibitory regulators are compatible with other necessary inhibitory regulators and any sufficient regulators.

**TABLE 2:** Compatibility of the causal logic implications of regulator nodes. The matrix lists the compatibility of different regulators with varying causal logic implications with ✓’s and ✗’s. The first row and the first column denote the logic implications of different regulators. A check (✓) entry denotes that the logic implications in the corresponding row title and column title are compatible while a cross (✗) entry denotes that they are incompatible.

Logic	Sufficient	Necessary	Sufficient inhibitory	Necessary inhibitory
Sufficient	✓	✗	✗	✓
Necessary	✗	✓	✓	✗
Sufficient inhibitory	✗	✓	✓	✗
Necessary inhibitory	✓	✗	✗	✓

#### 2.3 Resolving Apparently Incompatible Implications by Inferring New Relationships

**FIGURE 1:**
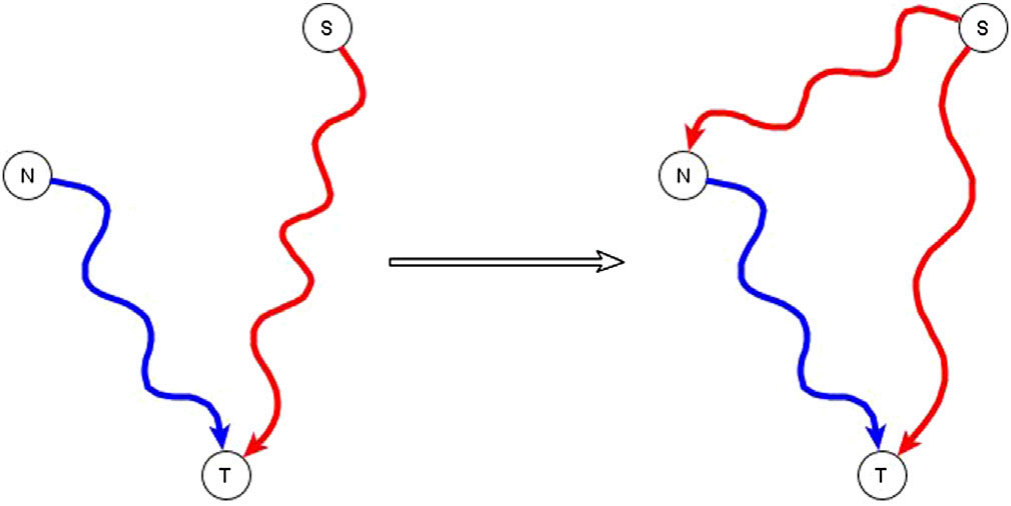
Illustration of the co-pointing subgraph theorem for inferring logic implication between two regulators. The source node S is sufficient indirectly (*via* a path or a subgraph) for the target node T and the non-source node N is necessary indirectly for the target node T. The two subgraphs from S to T and from N to T are co-pointing subgraphs. This leads to inference of a direct or indirect causal logic implication that the signal node S is sufficient for the non-source node N.

A subset of the incompatible relationships described in the previous subsection can be resolved if one or both of the apparently incompatible regulators is in reality an indirect regulator of the target node and if the two regulators are not independent of each other, but rather one of them has a logic implication on the other. This is expressed and proven in the co-pointing subgraph theorem of (Maheshwari and Albert, 2017). If a source node (S), i.e., a node with no regulators, is indirectly sufficient for a target node (T) and another node (N), which is not a source node, is directly or indirectly necessary for this target node, we say that there are two co-pointing subgraphs, one from S to T and one from N to T (Maheshwari and Albert, 2017)—see Figure 1. The co-pointing subgraph theorem from Maheshwari and Albert (2017) says that when there are two co-pointing subgraphs as in Figure 1, where source node S is sufficient and N is necessary to the target node, S must be sufficient for N. The simplest subgraph that satisfies this theorem is if the function of N is N* = S, and the function of the target is T*= S and N. Here we extend the applicability of this theorem to the situation in which S is not a source node and there is no path from N to S.

The co-pointing theorem can be used to resolve certain kinds of apparently incompatible logic implications of indirect regulators by inferring new edges. Situations like this happen often in genetic or pharmacological knockout experiments that aim to identify putative signal transduction mediators. If the experiment finds that the knockout of N disrupts the signal transduction process that initiates from signal S, we conclude that N is necessary for the target node. This might seem incompatible with the knowledge that the signal S is sufficient for the target but in fact it is consistent if N is a mediator of the pathway that establishes a sufficient relationship from S to the target node. Therefore, we infer that S is sufficient for N *via* an edge, a path, or a subgraph.

## 3 Results

### 3.1 Our Proposed Method of Boolean Model Inference From Causal Logic Implications of Edges

We first give a high-level description of our inference process, then describe the details of each step in separate subsections. Boolean network inference using the causal logic method starts with a compilation of information regarding the interactions and inferred causal influences between different components of the network. When a target node has multiple regulators, we classify their effect on the target into three categories: direct relationships, indirect relationships that likely do not share mediators with any other relationships, and indirect relationships that likely share mediators. The first two categories are represented as edges in the Boolean network while the third may be implemented by paths or subgraphs. We compile the edges incident on each node into Boolean functions that best preserve their logic implications, resolving any incompatibilities. Finally, we evaluate the implementation of the mediator-sharing indirect causal logic relationships by paths and subgraphs, and if necessary add edges to reflect them, again resolving any incompatibilities in the Boolean functions. A Python implementation of this method is available in the GitHub repository https://github.com/parulm/suff_necc.

#### 3.1.1 Distilling Biological Knowledge and the Results of Perturbation Experiments Into Logic Implications

The first step is to extract a library of information from experiments regarding the behavior of the system in normal and perturbed settings. This information is then organized as a list of causal influences and interactions, with more details indicated about each of the relationships whenever known. Each entry must include whether the interaction is promoting or inhibiting the target node, whether these relationships are direct (i.e., due to a single reaction or physical interaction) or indirect (mediated by other components) and the causal logic implication of the regulator on the target node (if known).

Certain types of biological information naturally lend themselves to causal representation. The causal effect associated with a biochemical reaction can readily be determined from the information that the presence of the reactant(s), together with the enzyme that catalyzes the reaction, leads to the production of the biomolecule that is the product of the reaction. Thus, in an enzyme-catalyzed reaction both the reactant(s) and the enzyme are necessary for the product. More generally, if an experimenter observes that the knockout of a node (regulator) leads to no (or below threshold) levels or activity of another node (target), one can conclude that the regulator node is necessary for the target. The “necessary” designation incorporates the assumption that the knockout of the source node would lead to the inactivity of the target in a different context as well. This assumption is widely made in the biological literature, as reflected by terms such as “necessary” and “required”. If an experimenter observes that the sustained presence or constitutive activity of a regulator leads to high activity of another node (target), one can conclude that the regulator is sufficient for the target node. Given the fact that *in vivo* biological experiments involve multiple components in addition to the pair whose relationship is studied, the noted sufficient or necessary implication are provisional, conditioned on the presence or absence of other components (known or unknown) that define the biological context. Additional evidence that characterizes these possibly hidden components may necessitate the revision of the initial characterization.

Other types of biological information are better represented as multi-node relationships. Specifically, many biological experiments involve perturbing putative mediators and comparing an input-output relationship in the perturbed and normal systems. In these experiments, there are three essential entities, the input, the output and the mediator. This usually results in statements (three-node causal implications) of the form “A promotes (B induces C)” [see (Albert et al., 2017; S.; Li et al., 2006) for examples of such statements and how they were used during model construction]. In general, each such statement immediately leads to two derived statements. The first is that “B induces C”, which usually implies that B is sufficient for C. The second statement is that “A promotes C”. The causal logic implication of this statement is obtained by looking at the experiment regarding node A. The most frequently observed case is that knockout of A leads to a drastically reduced activity of C (below-threshold); in this case we conclude that A is necessary for C. The role of node A could also be inhibitory, leading to a statement of the type “A inhibits (B induces C)”. The most frequently observed case is that constitutive activation of such inhibitory A leads to a below-threshold activity of C; in this case we conclude that A is a sufficient inhibitor of C.

A third regulatory relationship that can sometimes be inferred from a promoting or inhibiting three-node relationship depends on the use of the result on co-pointing subgraphs. If B affects C indirectly and there is no path from A to B, for certain types of causal logic of A on C we can infer that B regulates A according to a specific causal logic. We do this using the co-pointing subgraph theorem (Maheshwari and Albert, 2017). Given that B is sufficient for C, the co-pointing subgraph theorem applies for two causal logic implications of A on C. The first case is when A is necessary for C—we can conclude in this case that B is sufficient for A. The second case is when A is a sufficient inhibitor of C—then we can conclude that B is a sufficient inhibitor of A. However, if A is sufficient for C, no inference of any relationship between B and A can be made.

#### 3.1.2 Assigning a Boolean Function for Each Node and Resolving Incompatibilities

We break up three-node causal implications into pairwise implications and assign a logic implication to each pairwise relationship as described above. We then consider each node of the network along with its regulators and the corresponding causal logic implications and use that information to obtain the Boolean function for the node. In the following we describe the method of determining Boolean functions in detail.

If a target node (T) has a single regulator (R), there are two general cases: the regulator is either promoting or inhibiting. If the regulator is promoting, its Boolean function would be T* = R; and if the regulator is inhibiting, the Boolean function would be T* = not R. When a target node has multiple regulators, we classify their effect on the target into three categories: direct relationships, indirect relationships that likely do not share mediators with any other relationships, and indirect relationships that likely share mediators. The third category consists of regulators of the target node that are likely connected to the target node by a path or subgraph, and this path may involve other, more direct regulators of the target node. For each of these regulators we need to evaluate, on a case-by-case basis, whether or not an edge from the regulator to the target is needed.

**FIGURE 2:**
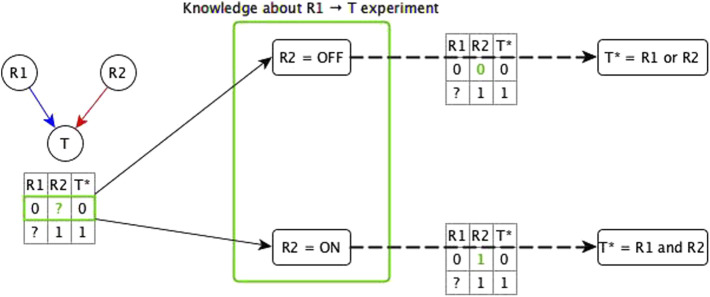
Illustration of the use of knowledge about the experimental setting to resolve incompatibility. A target node T has two regulators; one is concluded to be necessary (R1) from experiment/literature and the other is concluded to be sufficient (R2) from experiment/literature. The truth table below the network diagram shows these relationships: R1 → T necessary relationship means that when R1 is constitutively OFF, T stabilizes to OFF; R2 → T sufficient relationship means that when R2 is constitutively ON, T stabilizes to ON. Since this is an incompatibility, we consider two assumptions about the state of R2 (denoted by green “?”) in the experiment that concluded R1 to be necessary—the row marked in green in the truth table below the network. The first assumption is when R2 is OFF. In this case, there are two possible functions for T*: 1) T* = R1 and R2, and 2) T* = R1 or R2. If the rule were T* = R1 and R2, T would need to be OFF in the first row of the truth table, independent of the state of R1, hence, this leads us to conclude that the rule is T* = R1 or R2—with the corresponding truth table shown on the top dashed edge. When R2 = OFF, this rule becomes T* = R1, where R1 is indeed sufficient and necessary for T, making the “necessary” classification of R1 → T valid. The second assumption is when R2 is ON but T is OFF when R1 is OFF, which leads us to conclude that the rule is T* = R1 and R2 (see bottom dashed edge). When R2 = ON, this rule is reduced to T* = R1, where R1 is sufficient and necessary for T, making the “sufficient” conclusion about R1 → T valid. These two assumptions fall under the two templates of the dominant regulators resolution method. Due to commutativity of the Boolean operators, the resulting Boolean function would be the same whether the knowledge is about the R2 → T experiment or about the R1 → T experiment.

We start by considering the logic implications of direct relationships and indirect relationships that likely do not share mediators with any other relationships. In this case, there are two possibilities: all of the incoming logic implications are compatible, or the incoming edges have incompatible logic implications. In case of incompatible logic implications, we cannot directly define the Boolean function. These cases arise because the “sufficient” or “necessary” implication was premature and the target node’s activity in fact depends on the specific combination of regulators. The ideal resolution for these incompatible logic implications would be to do biological experiments that test both knockout and constitutive activation of each regulator (see Figure 2). This solution is often impossible to execute due to technical challenges and/or the intertwined nature of biological systems. Hence, we make use of two theoretical resolution methods. One is automated while the other requires manual curation.

The automated resolution method for incompatible logic implication is the dominant regulators method; this has two templates described as follows. One of these two templates considers sufficient regulators as dominant (i.e., if any of the sufficient regulators is active then the target node activates); the other considers necessary regulators as dominant (i.e., if any of the necessary regulators is inactive then the target node inactivates). This resolution method assumes that during the experimental result that concluded the logic implication that is incompatible with the dominant logic implication the dominant regulators were in their non-canalizing state. In the following we describe each template.

**FIGURE 3:**
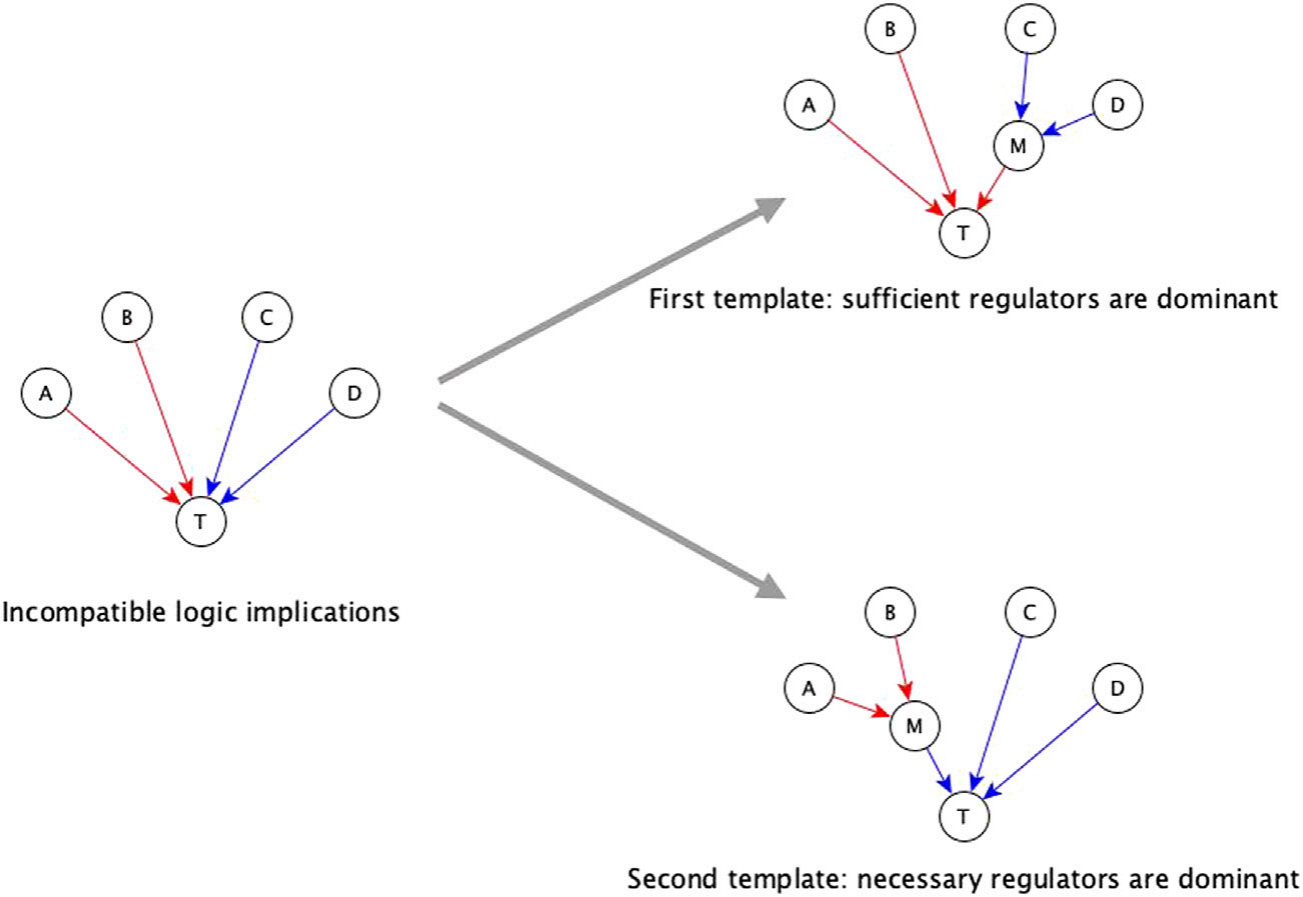
Dominant regulators method for the resolution of incompatible logic implications. Red edges indicate sufficient causal logic implication while blue edges indicate necessary causal logic implication. In this example, regulators A and B are sufficient while regulators C and D are necessary for the target node T—this is an incompatible combination of logic implications. The two templates for resolving this by the dominant regulators method are shown on the right. The top-right case shows the first template where sufficient regulators are considered to be dominant, hence, the necessary regulators are grouped together, and this group is marked as sufficient. In this case, node M is a mediator node that is sufficient for T. The Boolean rule for the target node is: T* = A or B or (C and D), which is equivalent to T* = A or B or M; where M* = C and D. The bottom-right case shows the second template where necessary regulators are considered to be dominant, hence, the group of sufficient regulators is marked as necessary. In this case, M is a mediator node that is necessary for T. The Boolean rule for the target node is: T* = (A or B) and C and D which is equivalent to T* = M and C and D; where M* = A or B.

The first automated template for resolving incompatibilities is to assume sufficient regulators are dominant. We impose this template by collecting all necessary regulators and marking them sufficient together, i.e., when all the necessary regulators are active, the target node will activate. Consider that target node T has regulators A, B, C, and D, where the edges A → T and B → T have sufficient logic implication while the edges C →T and D → T have necessary logic implication—see Figure 3. According to the first template, we group C and D together, resulting in the Boolean function T* = A or B or (C and D). When we group the regulators C and D together and mark them as sufficient together, we are implicitly assuming information about the states of the other regulators, i.e., A and B, during the experiments concerning C and D. Specifically, we are assuming that A and B are OFF during the experiment involving knockout of C (or knockout of D); in this context the experiment shows that C (or D) is necessary for T, in agreement with the Boolean rule obtained by the first template. Since necessary regulators are compatible with sufficient inhibitory regulators, they can also be grouped together with sufficient inhibitory regulators. In the above example, if the edge from C to T were instead sufficient inhibitory, the resulting Boolean function would be T* = A or B or (not C and D). Our code on the GitHub repository (https://github.com/parulm/suff_necc) lists the possible Boolean rules obtained by this automated method.

The second template in the automated resolution method is to give preference to the necessary logic implication and group the sufficient regulators. Going back to the example of Figure 3, the second template in this example (bottom right) leads to the Boolean rule T* = (A or B) and C and D. Since sufficient logic implication is compatible with necessary inhibitory logic implication, sufficient regulators can be grouped with necessary inhibitory regulators as well. In the previous example, if the edge from B to T was necessary inhibitory, the Boolean rule would be T* = (A or not B) and C and D. These resolution templates are meant for a quick construction of the Boolean function from incomplete information.

In the manual curation resolution method, whenever we come across incompatible logic implications, we further browse the literature to find information about the states (ON/OFF) of other nodes of the network during the experiment that was used to infer the logic implication. We then use this knowledge to pick the more likely of the two possibilities detailed in the previous paragraph. Often, there are more complex possibilities for the Boolean function, which we handle on a case-by-case basis. In many of these scenarios, we construct an incomplete truth table from the literature knowledge and combine it with common biological knowledge to obtain a Boolean function.

The automated and manual resolution methods can also be applied simultaneously—we can obtain the two templates from the automated method and pick one if it satisfies the existing knowledge and the findings from the literature. The manual curation method or the two methods used simultaneously will be more thorough than just using the automated method. However, the automated method can be more useful to identify cases that need manual attention, particularly when there are many incompatibilities. Also, in scenarios where there is no additional literature information available, the automated method is something to rely on. Regardless of the method, the resulting function is one of multiple possibilities compatible with the incomplete input information. The function needs to be subjected to experimental verification followed by improvement as necessary.

#### 3.1.3 Incorporating the Mediator-Sharing Indirect Relationships

**FIGURE 4:**
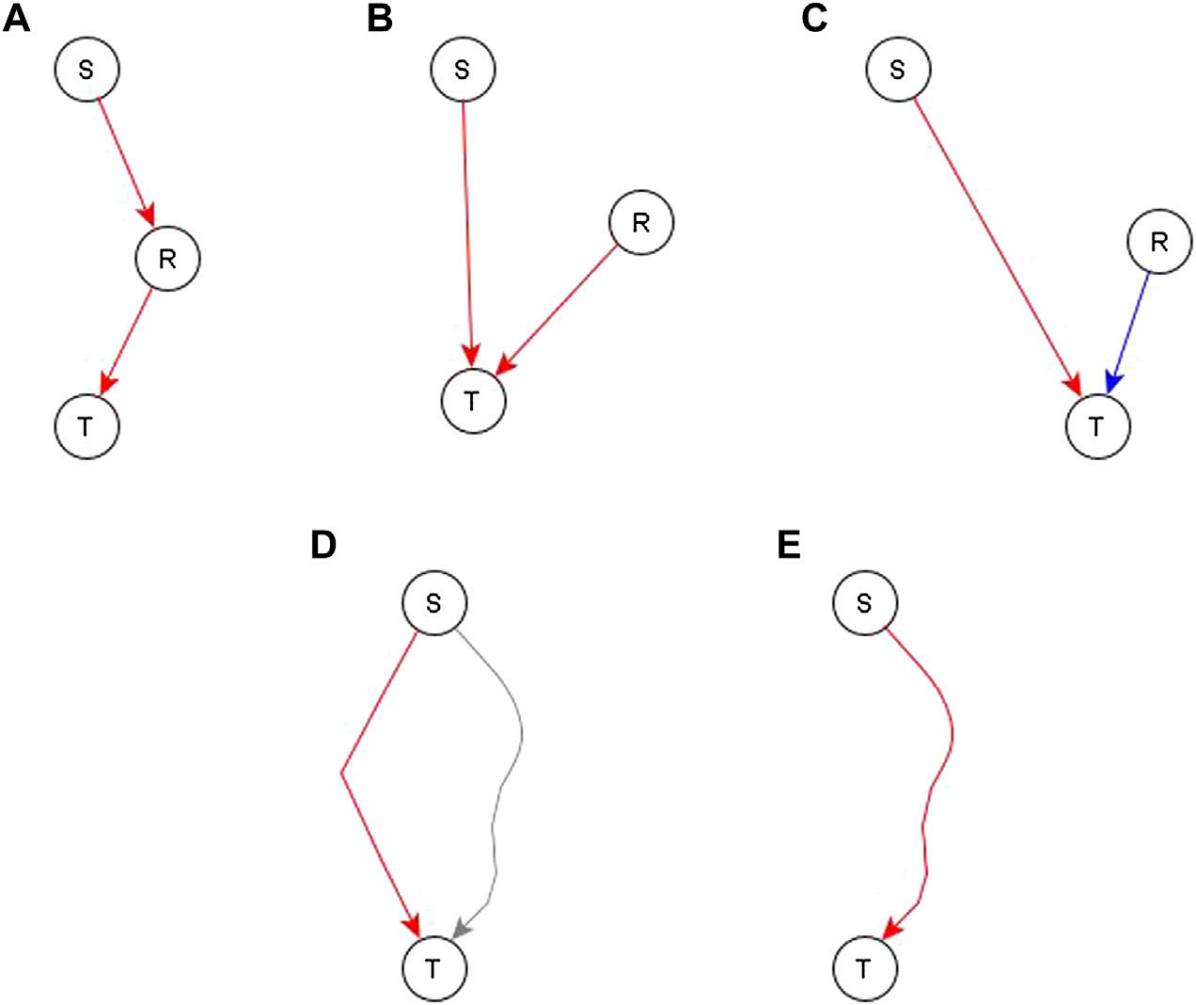
Different ways to incorporate an indirect relationship from a regulator S to a target node T when S is sufficient for T. Panels **(A–C)** describe the case when there is no existing path or subgraph from the regulator (S) to the target node (T) and panels D and E describe the case when there is an existing path/subgraph from S to T. **(A)**. An existing regulator of T, namely R, is sufficient for T. If there is also biological support for a pathway or causal relationship from S to R, we complete a sufficient path from S to T by adding a sufficient edge from S to R. **(B)**. An existing regulator R of T is sufficient for T but there is no evidence to support a causal relationship from S to R. In this case, we construct an independent sufficient edge from S to T. **(C)**. An existing regulator R is necessary for T. We cannot be confident that R does not influence S, thus the co-pointing theorem cannot be applied. Since the causal logic relationship between S and T is “sufficient”, we construct an independent sufficient edge from S to T. **(D)**. A path/subgraph exists from S to T, but its logic implication is not the same as the desired sufficient causal logic—this path is marked in gray. In this case, we add an independent sufficient edge from S to T to satisfy the “sufficient” logic. **(E)** A sufficient path/subgraph exists from S to T. In this case, the expected causal logic relationship already exists and hence we do not add any edges.

After a draft network is constructed from the direct and indirect but independent relationships between different nodes, we look at the evidence for the remaining indirect relationships. Specifically, we look at whether such relationships are reflected by paths or subgraphs with logic implications in the network. If no relevant path or subgraph is present, we add an edge to reflect the relationship—see panels A, B, and C of Figure 4. In some cases, an edge directed to one of the regulators of the target would complete a path or subgraph and thus would be more appropriate, as illustrated in Figure 4A for node S—most of these instances are handled on a case-by-case basis. In some other cases, this edge is pointing directly to the target node—this would mean that the process behind the relationship of S and T is independent of the other edges after all—this is illustrated in Figures 4B,C. There are two such cases, one where the addition of such an edge is logically compatible with other regulators so we just connect the newly added regulator with the dominant Boolean operator—illustrated in Figure 4B. The second case is where the edge is incompatible with the other regulators—illustrated in Figure 4C. In this case, we make the newly added regulator the dominant regulator and update the Boolean rule accordingly. In the case where a path/subgraph exists from the regulator to the target node, we have two cases. In the first case, the causal logic implication of the path/subgraph is not the same as the inferred causal logic implication of the new regulator. In this case, we add an edge from the newly added regulator to the target node—see Figure 4D. In the second case, the causal logic implication of the path/subgraph is the same as the inferred causal logic implication. In this case, we do not add a new edge—see Figure 4E.

Once all the experimental evidence is incorporated in the network, we use previously proposed logic reduction methods such as logic binary transitive reduction (l-BTR) to reduce this network and eliminate redundant edges (Albert et al., 2007). Logic binary transitive reduction consists of eliminating an edge from A to B if 1) it does not correspond to direct interactions and 2) there exists a path of the same sign and causal logic from A to B.

### 3.2 Application of the Network Inference Method to Biological Systems

#### 3.2.1 The ABA Induced Stomatal Closure Network

We illustrate the inference process on a signal transduction network that is known to be complex and for which a significant (but still incomplete) amount of causal evidence exists. The ABA induced stomatal closure network is a plant signaling network that illustrates the process of closing of the stomatal pores on the surface of leaves induced by the plant hormone abscisic acid (ABA). ABA is produced in the plant in response to drought or other desiccating stress. This stomatal closure process involves the interconnected and interdependent activities of many ion transport proteins, enzymes and other biomolecules. This process is important to study since the stomatal pores are responsible for intake of CO_2_ for photosynthesis and water loss in transpiration. In this case study, we build upon multiple previous studies on understanding this complex process by the means of a Boolean network model (Li, S. et al., 2006; Sun et al., 2014; Albert et al., 2017).

We do a careful analysis of the literature relevant to ABA induced stomatal closure. We derive pairwise relationships from three-node observations as described earlier. We find the associated causal logic corresponding to each pairwise relationship (see Supplementary Table S1). If the experiment reports strong qualitative results, we directly conclude the causal logic effect from there. There are two categories of such strong qualitative results. If the knockout of a gene (gene A) leads to a drastic decrease in the activity of the protein product of another gene (gene B), we conclude that gene A is necessary for gene B. If there is evidence of a reaction or physical interaction between the products of gene A and B, we mark the edge as direct; otherwise it is marked “not direct”. The second category is the observation that the supply of a molecule (X) leads to a drastic increase in the activity of a protein (Y), in such cases we conclude that X is sufficient for Y (directly or indirectly). In some cases, the causal logic implication has a lesser confidence (due to quantitative nuances or to expected combinatorial effects of multiple regulators), these cases are marked with an asterisk (*) in Supplementary Table S1. We take extra care in finding the Boolean functions in these cases as these relationships may actually be neither sufficient nor necessary. The relationship between the regulator D and target node T in the first template (top-right) of Figure 3 is an example of such a complex relationship. In the cases where it is applicable, we also use the result on co-pointing subgraphs to infer edges, see Supplementary Table S2. We use the causal logic implication we find to infer the Boolean network by our method. Here, we present selected cases that exemplify the inference method.

**Example of sufficient and necessary relationship**. ABA activates RCARs (Park et al., 2009). RCARs is a collective node representing the PYR/PYL family of proteins, which are soluble ABA receptors that directly bind to ABA. Their strict dependence on ABA leads us to conclude a sufficient and necessary relationship from ABA to RCARs. This is further reinforced by the necessary nature of RCARs in the stomatal closure process reported in Gonzalez-Guzman et al. (2012).

**FIGURE 5:**
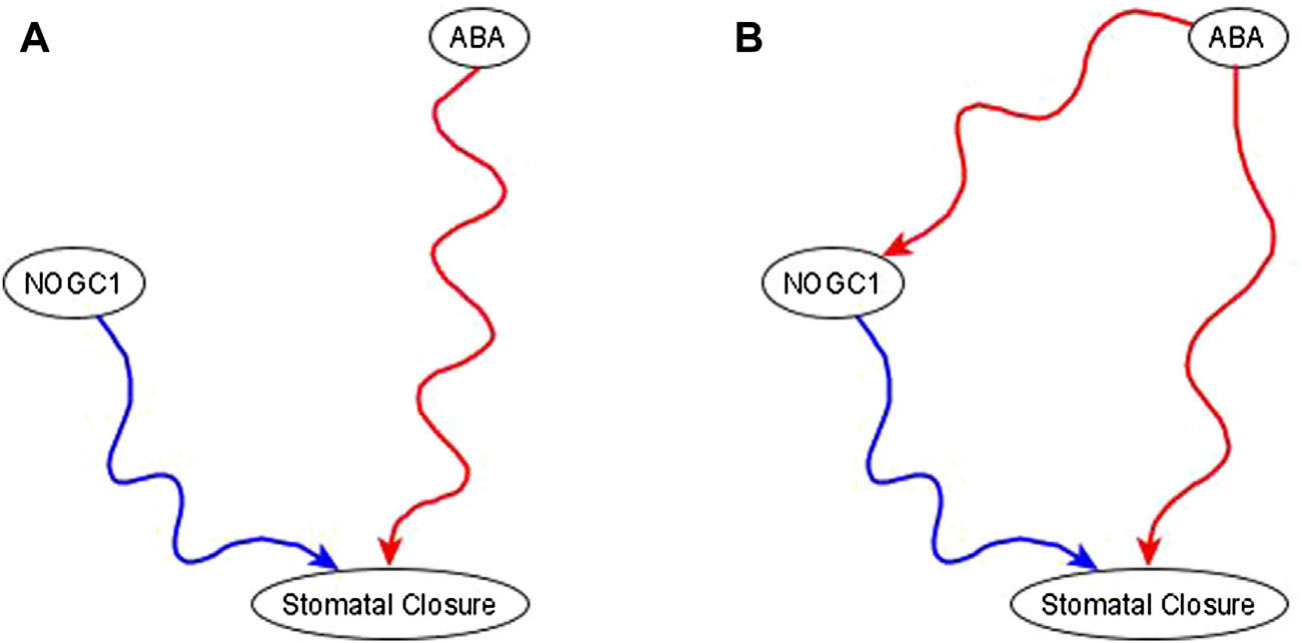
Use of co-pointing subgraph inference method in the ABA signaling network. The signal, and source node of the network, ABA, is well-known to be sufficient for the target node stomatal closure and Nitric Oxide dependent Guanylate Cyclase (NOGC1) is a putative mediator of the signaling process. **(A)**. Experimental results show that NOGC1 knockout leads to a higher stomatal aperture, i.e., NOGC1 KO prevents the closing of the stomata, implying that NOGC1 is necessary for closure. **(B)** As per the co-pointing subgraph theorem, ABA must be sufficient for NOGC1. In a previously reported Boolean model of ABA induced closure (Albert et al., 2017), there is indeed a sufficient relationship from ABA to NOGC1.

**Example of sufficient relationship**. SPHK1 and SPHK2 are sphingosine kinases denoted together by one node as SPHK1/2. Phosphatidic acid (PA) interacts with both SPHK1 and SPHK2 and upon binding, it increases the activity of SPHK1/2. An increase in concentration of PA leads to increase in activity of SPHKs 1 and 2 as reported in Figures 4, 5 of (Guo et al., 2011). Hence, we conclude the logic implication of the edge from PA to SPHK1/2 to be sufficient.

**Example of necessary relationship**. Ca^2+^_c_ promotes PLDα1 activity (Qin et al., 1997). Ca^2+^_c_ is required for the activation of the enzyme PLDα1. The analysis in Qin et al. (1997) shows that a reduction in the Ca^2+^ concentration leads to a reduction in the PLDα1 activity—see Figures 3, 4 of (Qin et al., 1997). We conclude that Ca^2+^_c_ is necessary for PLDα1 activity.

**Example of the use of co-pointing subgraphs** to characterize the indirect effect of ABA on nitric oxide-dependent guanylate cyclase (NOGC1). It is well-known that ABA is sufficient for stomatal closure (Joudoi et al., 2013; Albert et al., 2017). The results in Joudoi et al. (2013) show that knockout of *NOGC1* prevents stomatal closure (see Figure 2A of Joudoi et al. (2013). Hence, NOGC1 is necessary for closure. As its name indicates, NOGC1 is regulated by nitric oxide, thus it cannot be a source node. As per the co-pointing subgraph theorem (Maheshwari and Albert, 2017), this implies that ABA must be sufficient for NOGC1, which must be reflected by a sufficient path or subgraph in the resulting network (see Figure 5).

**FIGURE 6:**
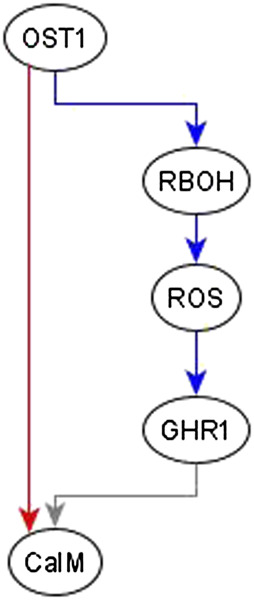
Addition of sufficient edge from OST1 to CaIM to reflect the causal logic inferred from the literature. The sufficient regulatory relationship of OST1 and CaIM is not reflected in the path given by OST1 → RBOH → ROS → GHR1 → CaIM. The path from OST1 to GHR1 is necessary but the edge from GHR1 to CaIM is neither sufficient nor necessary since the rule for CaIM is CaIM* = Actin Reorganization or (NtSyp121 and GHR1 and MRP5) or not ABH1 or not ERA1 or OST1. Hence, the total path from OST1 to CaIM does not have any logic implication. The sufficient edge from OST1 to CaIM is hence added. Edges in red color are sufficient, in blue color are necessary, and in gray color are neither sufficient nor necessary.

**Example of adding an edge from an indirect regulator.** When we observe an indirect regulator that already has a path to the target, we add an edge only if the path does not have the same logic implication—the case shown in Figure 4D. For example, the inference process is provided with the information that OST1 is sufficient for CaIM. There is already a path from OST1 to CaIM: OST1 → RBOH → ROS → GHR1 → CaIM, but the logic implication of this path is not “sufficient” and hence we add a sufficient edge from OST1 to CaIM. The resulting feed-forward loop is illustrated in Figure 6. As biological knowledge increases, this edge will likely be refined and populated by mediators or, refinement/correction of the existing path may render this edge unnecessary.

**FIGURE 7:**
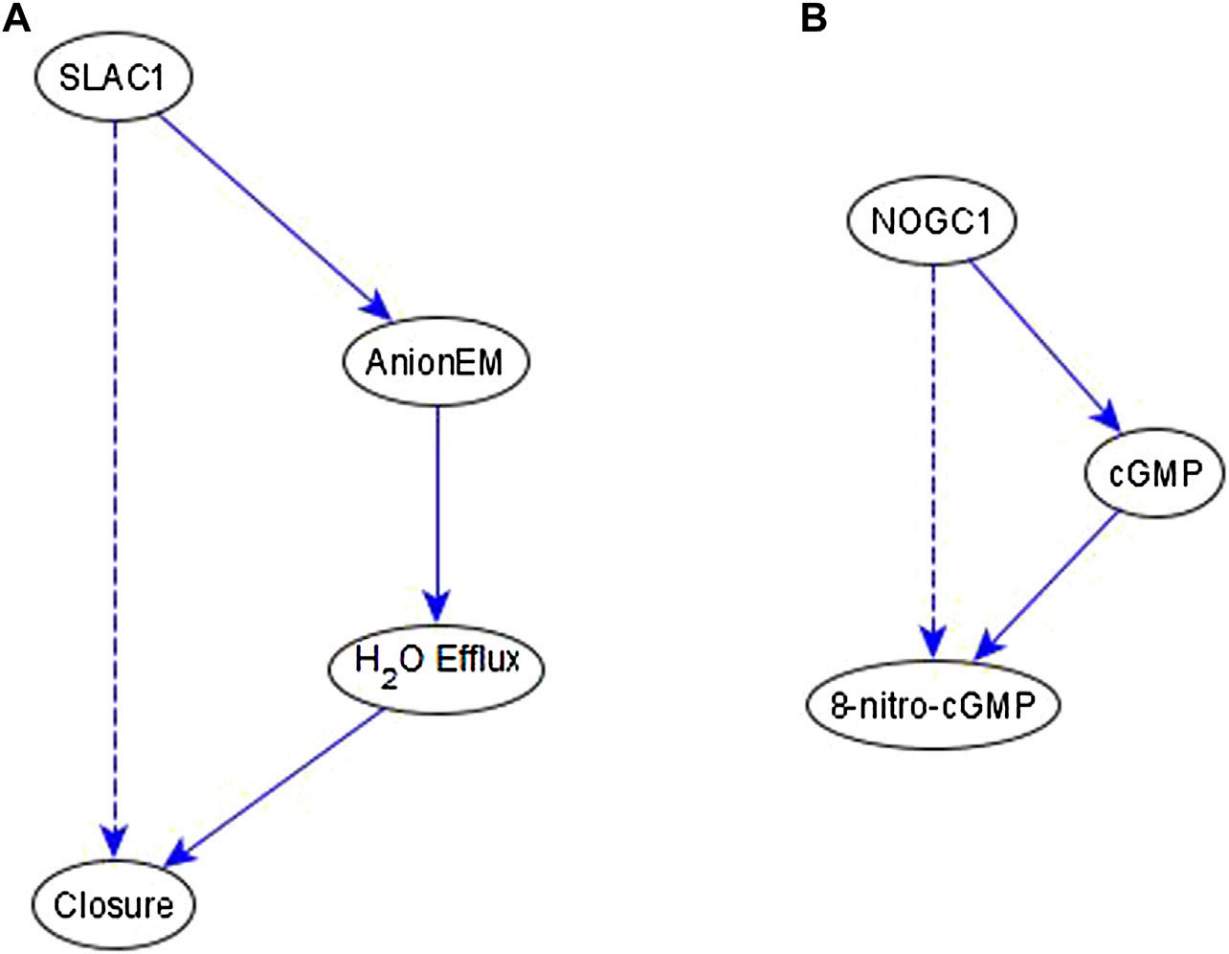
Example of indirect regulators with the inferred causal logic implication reflected in a path. **(A)**. SLAC1 is inferred to be a necessary regulator of Closure, which is reflected by the necessary path formed by SLAC1, AnionEM, H_2_O Efflux, and Closure. Hence the dashed edge from SLAC1 to Closure is not added to the network. **(B)** NOGC1 is inferred to be a necessary regulator of 8-nitro-cGMP, which is reflected by the necessary path formed by NOGC1, cGMP, and 8-nitro-cGMP. Hence the dashed edge from NOGC1 to 8-nitro-cGMP is not added to the network.

**Example of an indirect relationship reflected by a path/subgraph.** If an indirect regulator with a certain causal logic implication already has a path or subgraph to the target node with the same logic implication, we do not add an edge—case shown in Figure 4E. This happens frequently in the ABA network. Here, we produce two examples that illustrate this. In the first case, we infer a necessary regulation of stomatal closure by SLAC1 [an ion channel that mediates anion efflux (AnionEM)] from the experimental observation that *SLAC1* knockout disrupts 8-nitro-cGMP -induced stomatal closure as shown in Figure 10C of (Joudoi et al., 2013). As shown in Figure 7A, there already exists a path, SLAC1 → AnionEM → H_2_O Efflux → Closure, comprised of necessary edges. Hence, the logic implication is expressed by the path; we do not add the “necessary” edge from SLAC1 to Closure—shown as a dashed edge. In the second case, the input to our inference method indicates a necessary regulation of 8-nitro-cGMP by NOGC1—see Figure 7B. The path NOGC1 → cGMP → 8-nitro-cGMP is also necessary and hence we do not add the NOGC1 → 8-nitro-cGMP edge shown as a dashed edge.

The causal logic inference method is applied to 206 regulatory relationships collected in Supplementary Table S1 of (Albert et al., 2017), among which 107 relationships were known to be direct and 99 not known to be direct. An example application is available on the GitHub repository (see Methods). Among all these regulatory relationships, we could assign a logic implication to 196, of which 47 have a lower confidence (marked with an asterisk in Supplementary Table S1). We used the result on co-pointing subgraphs to infer 17 regulatory relationships, of which 8 resulted in the inference of a new edge. The remaining 9 cases corresponded to existing paths and subgraphs of the same logic implication. We then used the causal logic algorithm (Maheshwari and Albert, 2017) to look at the logic implications of the regulators for each node and construct the Boolean rules. In this process, 13 of the 62 nodes had incompatibility in the logic implications of the regulators. We used the dominant regulators method to resolve 7 of these cases. For the remaining 6 cases, we re-evaluated the causal logic implications and constructed the complete or incomplete truth table from data in the literature.

Our method, which only rarely needs manual interpretation and knowledge of the biology beyond the causal logic implication of an edge, re-discovered the Boolean rules of the ABA network correctly in 48 of 62 cases of inferring Boolean rules (see Supplementary Text S1). Following the second resolution method, we did an in-depth literature study for the remaining 14 cases. The methodology of this in-depth study involved constructing the incomplete truth table to find the exact rules. This methodology led to updated rules in 3 of the 14 cases, namely, PA (see Supplementary Table S3), AnionEM (see Supplementary Table S4), and OST1 (see Supplementary Table S5). Even after this update, only the rule for OST1 matched the previously reported rule (Albert et al., 2017). The remaining 13 discrepancies are marked in bold in Supplementary Text S1 and are explained in detail in Supplementary Text S2. We believe they can be best resolved with new experimental results, leading to higher confidence in one of the possible Boolean functions.

#### 3.2.2 The Network Corresponding to Epithelial to Mesenchymal Transition

As a second case study we consider another process whose underlying network is known to be complex: the epithelial to mesenchymal transition (EMT). Steinway et al. (2014) constructed a signal transduction network, and a Boolean model, whose outcome is the transcriptional downregulation of E-cadherin, which is a hallmark of EMT. As an additional test of our method we re-infer the Boolean model from a subset of the information that was used to construct the original model. We derive logical observations for every regulator—direct target pair from the Boolean functions of the EMT model, for a total of 127 edges (Steinway et al., 2014). We then modify this information to be more representative of characteristic use cases of the inference method. Specifically, we add indirect edges, or replace two-node paths by indirect edges, for a total of 18 changes to the input information. Some of these indirect edges correspond to a path of the same causal logic implication. Other indirect edges replace paths of the same causal implication. The logical observations used as inputs to the inference process are detailed in Supplementary Table S6. We use our inference method and find that it correctly resolves each modification.

**FIGURE 8:**
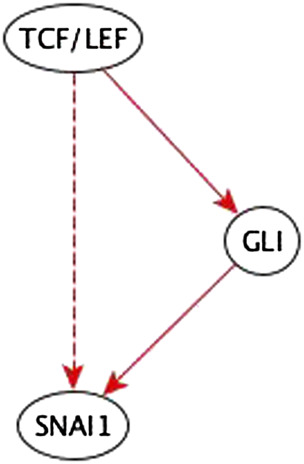
Example of an input perturbation where an indirect regulator has the same logic implication reflected by a path formed by direct regulators. TCF/LEF is a sufficient indirect regulator of SNAI1 and a sufficient direct regulator of GLI which is a sufficient direct regulator of SNAI1. Hence the sufficient indirect edge from TCF/LEF to SNAI1 is reflected in the sufficient path TCF/LEF → GLI → SNAI1.

**FIGURE 9:**
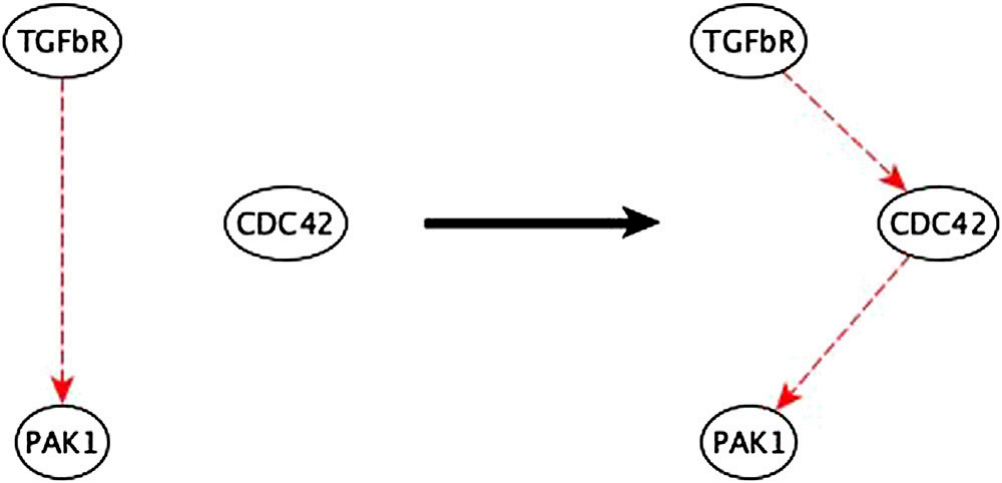
Example of mediator node inference. The sufficient indirect regulation of PAK1 by TGFβR can be mediated by CDC42 as a sufficient path.

**FIGURE 10:**
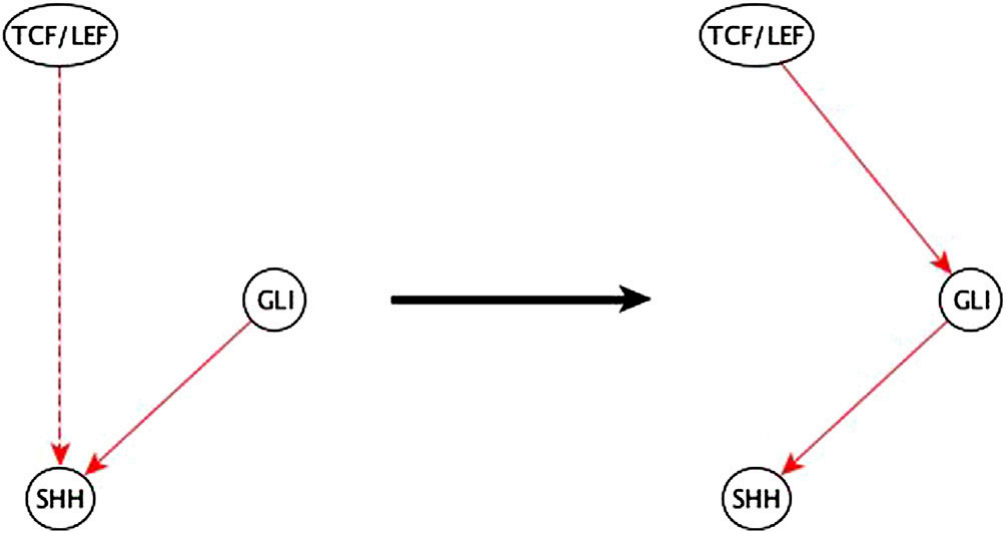
Example of half-known mediator node inference. The inference input information reveals that TCF/LEF is indirectly sufficient for SHH and that GLI is directly sufficient for SHH. This helps the biologist infer that the indirect sufficient regulation of SHH by TCF/LEF could be *via* GLI and potentially achieved by a TCF/LEF → GLI sufficient edge.

**FIGURE 11:**
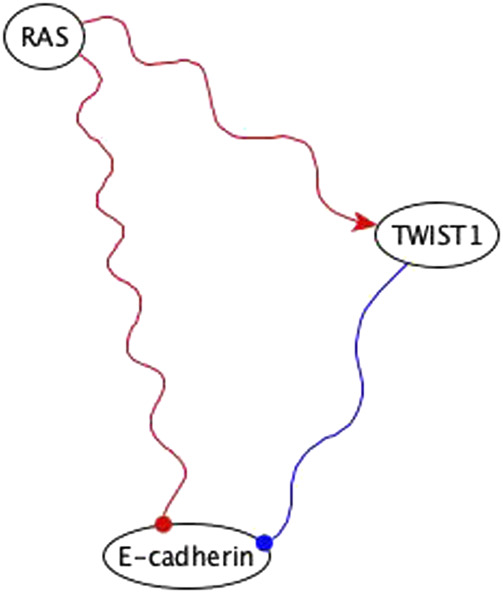
Example of using co-pointing theorem for inference in the EMT network. RAS is sufficient inhibitory for E-cadherin *via* a subgraph and TWIST is a necessary inhibitory for E-cadherin. Sufficient inhibitory and necessary inhibitory logic implications are not compatible but since they share the same regulator, we can use the co-pointing theorem to conclude that RAS must be sufficient for TWIST1, which will result in the elimination of the edge from RAS to E-cadherin in the final version of the network.

1. Edges that correspond to a path of the same causal logic implication are reduced during the inference process.
Example: The path TCF/LEF → GLI → SNAI1 is a sufficient path. So, the added TCF/LEF → SNAI1 sufficient indirect edge is redundant; it is reduced in the inference process – see Figure 8.
2. In cases where a two-node path is replaced by an indirect edge of the same logic implication, the inference method indicates potential mediator nodes, thus aiding the biologist in inferring the mediator – see Figure 9. We verified that all the suggested mediators were in fact actual mediators in the published EMT model.
3. A variant of the previous case is when a two-edge path between a regulator and a target is disrupted by deleting either the incoming or outgoing edge of the mediator node and is replaced by an indirect edge of the same logic implication. The inference method completes the path and infers a specific edge and logic implication for the mediator. This gives an even stronger aide for the biologist to infer the mediator compared to the previous case. An example of this case is listed in Figure 10.
4. The inference method used the co-pointing theorem to resolve discrepancies between incompatible indirect logic implications. An example is illustrated in Figure 11.

The Boolean functions resulting from the inference process are given in Supplementary Text S3. They are identical to the functions of the original EMT model.

To further test the accuracy and sensitivity of our method, we reduced the input information being provided to the code and assessed the accuracy of the resultant output. We provided ∼80% of the initial input information (see Supplementary Table S7) and found that the method correctly infers the Boolean function of 41 of the 59 nodes in the network (see Supplementary Text S8), i.e., ∼70% of the functions are correctly inferred.

## 4 Discussion

In this work, we present a combined Boolean network inference method that infers the network topology and the Boolean function for each node by assigning a causal logic implication to pairs of network components based on parsimonious interpretation of the results of perturbation experiments. This method significantly reduces the manual work needed to construct a Boolean network and infer its update rules. Our method not only eases the model construction process but also points to conflicting elements of the network which can thereafter be used to guide follow-up experiments and hence improve biological understanding. In certain stages of the model construction process we have more than one option for Boolean functions, which can lead to an in-depth re-examination of the interpretation of experimental results. This often provides specific relationships to search for in the literature that might have been missed in the initial scan.

In addition to indicating the knowledge gaps that need filling, this inference method can also give hints about the direct or indirect nature of relationships. For example, if a regulator is not known to be direct and the underlying causal logic relationship is found to be fulfilled by a path or a subgraph, we have reason to believe that this relationship is indirect, and we have a list of putative mediators to consider. This was shown in the specific case of Ca^2+^_c_ inhibition of PP2Cs in Maheshwari et al. (2019); the causal logic inference method makes it generally applicable.

Our application of this causal logic inference method to the well-studied ABA signaling process served as an excellent testbed for the method. A well-supported Boolean model of ABA-induced stomatal closure was reported in Albert et al. (2017), which we use to test the results of this inference method. The inferred Boolean functions of the ABA network (see Supplementary Text S1) particularly highlight the fact that this inference method gives a logical justification for choosing one of multiple possibilities in the face of insufficient knowledge. For example, the published and the inferred function for SLAH3 represent two different ways of resolving an incompatibility in the existing evidence; the inferred function is different from the published rule in Albert et al. (2017) on the basis of the stronger evidence of the sufficient inhibitory relationships between one of its regulators, ABI1, and SLAH3. Causal logic methodology working alongside other Boolean network analysis methods has helped us understand and improve the ABA network model (Maheshwari et al., 2019; Maheshwari et al., 2020). Despite the complexity of the network, we obtain promising results on this network using the causal logic inference method.

This method has limitations that should be addressed in future research. Any gaps or errors in the biological information used for inferring the causal logic could contribute to incorrect inferences. Furthermore, the assumption that a certain state of the regulator implies a state of the target node irrespective of the state of the other regulators does not always hold and instead the state of the target node is determined by a combination of the states of all regulators. Incompatibility between the regulators’ designations is an indicator of the inappropriateness of the causal implication. We proposed methods to resolve incompatibility by weakening the assumption and replacing it with multiple regulators being collectively sufficient or necessary. An undocumented regulator can also introduce incompatibility between the known regulators’ designations. Developing systematic methodologies to consider undocumented regulators will be the topic of future work. In cases of observed failure of the causal logic implication the fallback is to use manual inference from the collective experimental evidence, see for example the rule for OST1 in the ABA network (see Supplementary Table S5).

We envision the use of this method as one step in the cycle between experiment and modeling: its use speeds up the construction of an initial parsimonious Boolean model and allows more effort to be dedicated to experimental verification of the model and to the resulting model improvement. Future applications of this method for the inference of other signal transduction or gene regulatory networks will help us further refine this theory to further decrease the manual interpretation required to obtain the Boolean functions. We also believe that one can expand the causal logic inference method to multi-level discrete networks, as have been constructed for stomatal response (Sun et al., 2014; Gan and Albert, 2016). In these networks, each biomolecule has multiple levels, for example, 0, 1, and 2, and each level is represented by an individual node that has corresponding Boolean functions for different levels of the regulator nodes. “Necessary” can be extended to mean that the lowest level of the regulator, i.e., when the regulator is inactive, implies the lowest level of the target, i.e., the inactivity of the target, and “sufficient” can be extended to mean that the highest level of the regulator implies the highest level of the target. Criteria for identification of the group of nodes that together are sufficient can be derived in various modeling frameworks, e.g., in threshold models a node may be activated if two out of its three possible activators are present.

## Data Availability

The original contributions presented in the study are included in the article/Supplementary Material, further inquiries can be directed to the corresponding authors.
