# Supplementary material for: Inference of a Boolean Network From Causal Logic Implications
Source: Front Genet. 2022 Jun 16;13:836856. doi: 10.3389/fgene.2022.836856 (PMC9246059; doi:10.3389/fgene.2022.836856)
Supplement: Supplementary file 1 [file Presentation1.pdf]

Supplementary File

Inference of a Boolean Network from Causal Logic Implications

Parul Maheshwari, Sarah M. Assmann, Reka Albert

This file contains Supplementary Tables S1-S7 and Supplementary Text S1-S5.

Supplementary Table S1. List of interactions and their causal logic implications pertaining to ABA induced stomatal closure.

The published data in (1) reports 206 regulatory relationships with literature references for each of the relationships. We review the literature references to find the causal logic implication of each of the regulations. These causal logic implications fall into six types: sufficient (s), necessary (n), sufficient inhibitory (si), necessary inhibitory (ni), sufficient and necessary (sn), and sufficient and necessary inhibitory (sni). This table lists the regulatory relationship, the nature of the effect: promoting or inhibiting, the type of interaction: direct or not direct, the corresponding references and the causal logic implication. The logic implication marked with an asterisk (*) shows a lower confidence in the logic implication compared to the ones without an asterisk. The last column is relevant to indirect causal relationships only and indicates whether there is at least circumstantial evidence that this relationship is independent from (not mediated by) other regulators of the target node (node B).

Node A (regulator)	Node B (target)	Effect	Int. type	Ref	logic	Likely independent?
8-Nitro-cGMP	ADPRC	Promotes	Not direct	(2)	sn	Yes
ABA	RCARs	Promotes	Direct	(3–6)	sn	N/A
ABA	PEPC	Inhibits	Not direct	(7)	si	Yes
ABA	PI3P5K	Promotes	Not direct	(8)	s	Yes
ABA	SPHK1/2	Promotes	Not direct	(9–12)	s	No
ABA	AtRAC1	Inhibits	Not direct	(13)	ni	Yes
ABA	Malate	Inhibits	Not direct	(14)	si	No
ABH1	CaIM	Inhibits	Not direct	(15)	ni	Yes
ABI	AtRAC1	Promotes	Not direct	(13)	s	Yes

ABI	SLAH3	Inhibits	Direct	(16)	si	N/A
ABI1	SLAC1	Inhibits	Direct	(17)	si	N/A
ABI1	OST1	Inhibits	Direct	(4,18,19)	si	N/A
ABI2	OST1	Inhibits	Direct	(19)	si	N/A
HAB1	OST1	Inhibits	Direct	(19)	si	N/A
ABI2	GHR1	Inhibits	Direct	(20)	si	N/A
ABI2	SLAC1	Inhibits	Direct	(17–21)	si	N/A
Actin reor-ganization	CaIM	Promotes	Not direct	(22)	s	Yes
ADPRc	cADPR	Promotes	Direct		n	N/A
AGB1	AGG3	Binds	Direct	(23)	sn	N/A
AnionEM	Depolarization	Promotes	Direct	(24)	s	N/A
AnionEM	Malate	Inhibits	Direct	(25,26)	si	N/A
AnionEM	H_2O Efflux	Promotes	Not direct		n	Yes
ARP2/3 complex	Actin reorganization	Promotes	Direct	(27,28)	n	N/A
AtMPK9 (MPK9/12 node)	AtMPK9	Promotes	Direct	(29)	s	N/A
AtRAC1	Actin reorganization	Inhibits	Not direct	(13)	si	Yes
AtSPP1	S1P	Inhibits	Direct	(30)	si	N/A
Ca^{2+}	pH_c	Promotes	Not direct	(31)	n*	No
Ca^{2+}	TCTP	Promotes	Direct	(32)	s	N/A
Ca^{2+}	Ca^{2+} ATPase	Promotes	Direct	(33)	sn	N/A
Ca^{2+}	KEV	Promotes	Not direct	(34)	s	Yes
Ca^{2+}	H^+ ATPase	Inhibits	Direct	(35)	si	N/A
Ca^{2+}	Depolarization	Promotes	Direct	(36)	s	N/A
Ca^{2+}	CPK3 and CPK21 (CPK3/21 node)	Promotes	Direct	(37)	s	N/A
Ca^{2+}	PLDα1 (PLDα node)	Promotes	Direct	(38)	n	N/A
Ca^{2+}	PLC	Promotes	Direct	(39)	n	N/A
Ca^{2+}	V-ATPase	Promotes	Not Direct	(40)	s	Yes
Ca^{2+} ATPase	Ca^{2+}	Inhibits	Direct	(33)	si	N/A
cADPR	CIS	Promotes	Not Direct	(41,42)	s	Yes
CaIM	$Ca^{2+}{}_c$	Promotes	Direct	(43–46)	s	N/A
cGMP	8-nitro-cGMP	Promotes	Direct	(2)	n	N/A
CIS	$Ca^{2+}{}_c$	Promotes	Direct	(47,48)	s*	N/A
CPK21	SLAC1	Promotes	Direct	(21)	n*	N/A
CPK23	SLAC1	Promotes	Direct	(21)	n*	N/A
CPK23	SLAH3	Promotes	Direct	(16,37)	s*	N/A
CPK3/21	SLAC1	Promotes	Direct	(21,37,49)	s	N/A
CPK3/21	SLAH3	Promotes	Direct	(16,37)	s	N/A

CPK3/21	CPK3/21	Promotes	Direct	(50)	s	N/A
CPK6	SLAC1	Promotes	Direct	(37,49,51)	s	N/A
CPK6	SLAH3	Promotes	Direct	(37)	s	N/A
DAG	PA	Promotes	Direct	(52)	n*	N/A
DAGK	PA	Promotes	Direct	(52)	n*	N/A
Depolarization	KOUT	Promotes	Direct	(53)	n	N/A
ERA1	ROP10	Promotes	Not direct	(54)	n	Yes
ERA1	CaIM	Inhibits	Not direct	(55)	ni	No
GAPC1 and GAPC2 (GAPC1/2)	PLDδ	Promotes	Direct	(56)	n	N/A
GCR1	GPA1	Inhibits	Direct	(57)	ni	N/A
GEF1, GEF4, GEF10 (GEF1/4/10 node)	ROP11	Promotes	Direct	(58,59)	s	N/A
GHR1	SLAC1	Promotes	Direct	(20)	s*	N/A
GPA1	AGB1	Binds	Direct, undirected	(60)	sn	N/A
GPA1	PLDα1	Promotes	Direct	(61)	n*	N/A
GTP	cGMP	Promotes	Direct	(62)	n	N/A
H^+ ATPase	Depolarization	Inhibits	Direct	(63,64)	si*	N/A
InsP3	CIS	Promotes	Not direct	(48)	s	No
InsP3	InsP6	Promotes	Direct	(65)	sn	N/A
InsP6	CIS	Promotes	Not direct	(47)	s	No
K^+ efflux	H_2O efflux	Promotes	Not direct	(53)	n	Yes
K^+ efflux	Depolarization	Inhibits	Direct	(53)	si*	N/A
KEV	K^+ efflux	Promotes	Direct	(34)	n	N/A
KEV	Depolarization	Promotes	Direct	(34)	s*	N/A
KOUT	K^+ Efflux	Promotes	Direct	(53)	n	N/A
Malate	H_2O efflux	Inhibits	Not direct	(14)	si*	No
Microtubule depolymerizati on	Stomatal closure	Promotes	Not direct	(66)	n	Yes
Microtubule depolymerizati on	Microtubule depolymerization	Promotes	Direct	(67)	s	N/A
NAD^+	cADPR	Promotes	Direct	(68)	n	N/A
NADPH	ROS (H_2O_2)	Promotes	Direct		n	N/A
NADPH	NO	Promotes	Direct	(69)	n	N/A
NIA1/2	NO	Promotes	Direct	(69)	n	N/A
Nitrite	NO	Promotes	Direct	(69)	n	N/A
NO	NOGC1	Promotes	Direct	(62)	s	N/A
NO	8-Nitro-cGMP	Promotes	Not direct	(2)	n	No
NO	KOUT	Inhibits	Direct	(70)	si	N/A
NOGC1	cGMP	Promotes	Direct	(62)	n	N/A

NtSyp121-Sp2	CaIM	Inhibits	Not direct	(71)		No
OST1	SLAC1	Promotes	Direct	(17,72)	n	N/A
OST1	QUAC1	Promotes	Direct	(26,73,74)	n	N/A
OST1	RbohD/F (RBOH)	Promotes	Direct	(46,75–77)	n	N/A
OST1	PIP2;1	Promotes	Direct	(78)	s	N/A
PA	ABI1	Inhibits	Direct	(79,80)	si	N/A
PA	SPHK1 and SPHK2 (SPHK1/2)	Promotes	Direct	(12)	s	N/A
PA	RBOH	Promotes	Direct	(81)	n	N/A
PC	PA	Promotes	Direct	(38)	n*	N/A
PEPC	Malate	Promotes	Not direct	(7)	n	Yes
pH_c increase	KOUT	Promotes	Not direct	(82)		No
pH_c increase	H^+ ATPase	Inhibits	Not direct	(83)	si	No
pH_c increase	ABI1	Promotes	Direct	(84)	n	N/A
pH_c increase	pH_c	Inhibits	Not direct	(63,85,86)		No
PI3P5K	PtdIns(3,5)P2	Promotes	Direct	(8)	sn	N/A
PIP2;1	H_2O Efflux	Promotes	Direct	(78)	n	N/A
PLC	DAG	Promotes	Direct		n	N/A
PLC	InsP3	Promotes	Direct		n	N/A
PLC	PtdIns(4,5)P2 (PI4P5P2)	Promotes	Direct	(87)	s*	N/A
PLDα	PA	Promotes	Direct	(80)	n*	N/A
PLDδ	PA	Promotes	Direct	(88)	n*	N/A
PP2CA	SLAC1	Inhibits	Direct	(89)	si	N/A
PP2CA	OST1	Inhibits	Direct	(89)	si*	N/A
PtdIns(3,5)P2	V-PPase	Promotes	Direct	(8)	n	N/A
PtdIns(4,5)P2	DAG	Promotes	Direct		n	N/A
PtdIns(4,5)P2	InsP3	Promotes	Direct		n	N/A
PtdInsP3	Actin reorganization	Promotes	Not direct	(90)	n	No
PtdInsP4	Actin reorganization	Promotes	Not direct	(90)	n	No
PtdInsP4	PtdIns(4,5)P2	Promotes	Direct	(87)	sn	N/A
QUAC1	AnionEM	Promotes	Direct	(25,26)	n*	N/A
RBOH	ROS	Promotes	Direct	(91)	n	N/A
RCARs	ABI1/ABI2/HAB1	Inhibits	Direct	(3,4,6)	si	N/A
RCARs	PP2CA	Inhibits	Direct	(92)	si	N/A
ROP11	ABI2	Promotes	Direct	(58)	s*	N/A
ROP11	ABI1	Promotes	Direct	(93,94)	s*	N/A
ROS	8-Nitro-cGMP	Promotes	Not direct	(2)	n	No
ROS	KOUT	Inhibits	Not direct	(95)	si	No

ROS	H^+ ATPase	Inhibits	Not direct	(96)	si	No
ROS	ABI1	Inhibits	Direct	(97)	si	N/A
ROS	HAB1	Inhibits	Direct	(98)	si	N/A
ROS	ABI2	Inhibits	Direct	(99)	si	N/A
S1P/PhytoS1P	S1P/PhytoS1P	Inhibits	Not direct	(9,63,85)	si*	No
SCAB1	Actin reorganization	Promotes	Direct	(100)	n*	N/A
SLAC1	AnionEM	Promotes	Direct	(17,25)	s	N/A
SLAH3	AnionEM	Promotes	Direct	(16,25)	n*	N/A
Sph	S1P/phytoS1P	Promotes	Direct	(9)	n	N/A
SPHK1/2	S1P	Promotes	Direct	(12,101)	n	N/A
TCTP	Microtubule depolymerization	Promotes	Direct	(32)	s	N/A
Vacuolar acidification	pH_c	Promotes	Direct	(8)	n	N/A
V-ATPase	Vacuolar acidification	Promotes	Direct	(8,102,103)	n	N/A
V-PPase	Vacuolar acidification	Promotes	Direct	(8)	n	N/A
8-Nitro-cGMP	Stomatal closure	Promotes	Not Direct	(2)	s*	No
ABA	Actin reorganization	Promotes	Not direct	(13)		No
ABA	8-Nitro-cGMP	Promotes	Not direct	(2)	sn	No
ABA	ROS	Promotes	Not direct	(104)		No
ABA	NO	Promotes	Not direct	(105,106)		No
ABA	K^+ ion release from vacuole (KEV)	Promotes	Not direct	(107,108)	n*	No
ABA	pH_c increase	Promotes	Not direct	(87)		No
ABA	H^+ ATPase	Inhibits	Not direct	(96)		No
ABA	Microtubule Depolymerizatio n	Promotes	Not direct	(109)	s	No
ABA	Vacuolar acidification	Promotes	Not direct	(8)	s*	No
Ca^{2+}_c	Stomatal closure	Promotes	Not direct	(110)		No
Ca^{2+}_c	SLAC1	Promotes	Not direct	(49,72,111,1 12)	s	No
NO	Closure	Promotes	Not direct	(113)		No
NO	cGMP	Promotes	Not direct	(114)	n*	No
NOGC1	8-nitro-cGMP	Promotes	Not direct	(2)	n*	No
NtSyp121-Sp2	Ca^{2+} transient	Inhibits	Not direct	(71)	si*	No
OST1	CaIM	Promotes	Not direct	(46)	s	No
pH_c increase	Vacuolar acidification	Promotes	Direct	(8)	n	N/A

PP2CA	Stomatal closure	Inhibits	Not direct	(115)	ni	No
ROS	NO	Promotes	Not direct	(106)	s	No
ROS	Microtubule Depolymerizatio n	Promotes	Not Direct	(109)	s*	No
ROS	Stomatal closure	Promotes	Not Direct	(20,116,117)	s	No
S1P	Stomatal closure	Promotes	Not direct	(9,118)	n*	No
ABI1	ABA induced pH_c increase	Inhibits	Not direct	(31)	si	No
ABI1	ABA activation of RBOH	Inhibits	Not direct	(119)	si	No
ABI1	SLAC1	Inhibits	Direct	(21)	si	N/A
ABI1	CPK3 and CPK6 activation of SLAC1	Inhibits	Direct	(37,51)	si	N/A
ABI2	ABA induced pH_c increase	Inhibits	Not direct	(31)	si	No
ABI2	CPK6 and CPK23 activation of SLAC1	Inhibits	Direct	(21,51)	si	N/A
AtSPP1	ABA-induced stomatal closure	Inhibits	Not direct	(30)	ni	No
TCTP	Ca^{2+}-mediated stomatal closure	Promotes	Not Direct	(32)	s	No
TCTP	ABA-mediated stomatal closure	Promotes	Not Direct	(32)	s	No
Ca^{2+}	S1P activation of PLDα	Promotes	Not direct	(101)	n*	No
Ca^{2+}	8-nitro-cGMP-mediated stomatal closure	Promotes	Not Direct	(2)	n*	No
Ca^{2+}	ABA induction of KEV	Promotes	Not direct	(108,120)	n	No
Ca^{2+}	ABA-induced NO production	Promotes	Not direct	(105)	n*	No
cADPR	8-nitro-cGMP-induced stomatal closure	Promotes	Unknown	(2)	n*	No
CIS	ABA-induced stomatal closure	Promotes	Not direct	(121)	n*	No
GAPC1, GAPC2	ROS activation of PLDδ	Promotes	Direct	(56)	s*	N/A
GHR1	ROS activation of CaIM	Promotes	Not direct	(20)	n	No
GHR1	Activation of SLAC1 by ROS	Promotes	Not Direct	(20)	n	Yes
GPA1	ABA activation of RBOH	Promotes	Not direct	(117)	n	No

GPA1	S1P – induced closure	Promotes	Not direct	(9)	n*	No
GPA1	ABA-induced CaIM	Promotes	Not direct	(117)	n	No
MPK9/12	ROS-mediated stomatal closure	Promotes	Not direct	(122)	n*	No
MPK9/12	Ca^{2+} induced activation of SLAC1	Promotes	Not direct	(122)	n	Yes
MRP5	ABA activation of CaIM	Promotes	Not direct	(123)	n	No
MRP5	Ca^{2+} activation of SLAC1	Promotes	Not direct	(123)	n	No
NIA1 and NIA2 (NIA1/2)	ROS induced NO production	Promotes	Not direct	(106)	n*	Yes
NO	NtSyp121-Sp2-mediated inhibition of ABA-induced stomatal closure	Inhibits	Not Direct	(71)	si*	No
NOGC1	ABA-induced stomatal closure	Promotes	Not direct	(2)	n*	No
NOGC1	NO-induced stomatal closure	Promotes	Not direct	(2)	n*	No
NtSyp121-Sp2 fragment (SNARE protein)	ABA-induced stomatal closure	Inhibits	Not Direct	(71)	si*	No
OST1	ABA induced pH_c increase	Promotes	Not direct	(31)	n*	No
H^+ ATPase	ABA-promotion of stomatal closure	Inhibits	Not direct	(64)	si*	No
pH_c increase	ABA activation of RBOH	Promotes	Not Direct	(86)	n	No
pH_c increase	ABA activation of SLAC1	Promotes	Not Direct	(124)	n	No
pH_c increase	ABA-promotion of NO production	Promotes	Not direct	(105)	n*	No
PLC	NO-mediated stomatal closure	Promotes	Not direct	(113)	n*	No
PLC	NO-mediated PA production	Promotes	Not direct	(113)	n*	No
PLDδ	NO induced stomatal closure	Promotes	Not direct	(125)	n	No
PLDδ	ROS induced stomatal closure	Promotes	Not direct	(56,125)	n*	No
PP2CA	CPK6-mediated activation of SLAC1 activity	Inhibits	Direct	(51)	si	N/A

PtdIns(3,5)P2	ABA-induced vacuolar acidification	Promotes	Not direct	(8)	n	No
PtdIns(3,5)P2	ABA-induced stomatal closure	Promotes	Not direct	(8)	n	No
PtdInsP3	ABA induced ROS production	Promotes	Not direct	(90,126,127)	n	No
QUAC1	Ca^{2+} induced closure	Promotes	Not direct	(74)	n	No
RBOH	ABA induced NO production	Promotes	Not direct	(106)	n	No
RCN1	ABA induced ROS production	Promotes	Not direct	(91,128)	n*	No
S1P	ABA-induced Ca^{2+} increase	Promotes	Not direct	(118)	n*	No
SLAC1	8-nitro-cGMP - induced stomatal closure	Promotes	Not direct	(2)	n*	No
SPHK1/2	ABA-induced stomatal closure	Promotes	Not direct	(10,11)	n	No
SPHK1/2	ABA activation of PLDα	Promotes	Not direct	(12)	n	No

Supplementary Table S2. List of inferred edges in ABA induced closure

We use the causal logic analysis result on co-pointing subgraph to infer an edge. This table lists all the regulations that can be inferred using co-pointing subgraphs. Some of these regulations were already reflected in the network from direct regulations, and hence we do not add a new edge in those cases. In the remaining cases, we infer a new edge and add it to the network. The first column lists the regulator of the inferred regulation, the second column gives the target node, the third column gives the logic. The logic implication is either of sufficient (s), necessary (n), sufficient inhibitory (si), necessary inhibitory (ni), sufficient and necessary (sn), sufficient and necessary inhibitory (sni). The fourth column gives the references for the regulation/interaction that led to the inference. The fifth column gives the regulation observed from the references that can be used in the format of co-pointing subgraphs. The sixth column lists whether a new edge was added or not. A new edge is not added when a logically equivalent path or subgraph already exists. In the cases where a new edge is not added, i.e., the sixth column entry is “no”, the seventh column lists the equivalent path. The last column indicates whether there is support for the inferred relationship being independent from other regulators of the target node.

**regulator**	**target**	**logic**	**Ref**	**original interaction**	**new edge**	**equivalent path**	**Likely independent?**
ROS	PLDδ	n	(56,125)	PLDδ is necessary for ROS induced stomatal closure	Yes		No
ROS	GHR1	s	(20)	GHR1 is necessary for ROS activation of CaIM/SLAC1	Yes		No
S1P	GPA1	s	(9)	GPA1 is necessary for S1P-induced closure	Yes		Yes
Ca^{2+}	MPK	s	(122)	MPK is necessary for Ca^{2+} induced activation of SLAC1	Yes		No
ROS	NIA1/2	s	(106)	NIA1/2 is necessary for ROS induced NO production	Yes		Yes
Ca^{2+}	QUAC1	s	(74)	QUAC1 is necessary for Ca^{2+} induced closure	Yes		No
ABA	ABI1	si	(31,119)	ABI1 is a sufficient inhibitor of ABA-induced pHc increase/RBOH activation	No	ABA -> RCARs -| ABI1	No
ABA	ABI2	si	(31)	ABI2 is a sufficient inhibitor of ABA-induced pHc increase/RBOH activation	No	ABA -> RCARs -| ABI2	No
ABA	Ca^{2+}	s	(105,108,120)	Ca2+ is necessary for ABA-induced KEV/NO production	No	ABA -> CIS -> Ca^{2+}	No

8-nitro-cGMP	cADPR	s	(2)	cADPR is necessary for 8-nitro-cGMP-induced stomatal closure	No	8-nitro-cGMP -> ADPRc -> cADPR	No
ABA	CIS	s	(122)	CIS is necessary for ABA-induced stomatal closure	Yes		No
ABA	GPA1	s	(117)	GPA1 is necessary for ABA activation of RBOH/CaIM	No	ABA -> SPHK1/2 -> S1P -> GPA1	No
NO	NOGC1	s	(2)	NOGC1 is necessary for NO induced stomatal closure	No	NO -> NOGC1	Yes
NO	PLDδ	s	(125)	PLDδ is necessary for NO induced stomatal closure	Yes		No
ABA	PtdIns(3,5)P2	s	(8)	PtdIns(3,5)P2 is necessary for ABA induced Vacuolar Acidification/ stomatal closure	No	ABA -> PI3P5K -> PtdIns(3,5)P2	No
ABA	S1P	s	(118)	S1P is necessary for ABA-induced Ca^{2+} increase	No	ABA -> SPHK1/2 -> S1P	No
ABA	SPHK1/2	s	(10–12)	SPHK1/2 is necessary for ABA induced PLDδ/stomatal closure	No	ABA -> SPHK1/2	No

Supplementary Table S3. Truth table for the regulator function of PA

Truth table extracted from Fig 1F of (88). The asterisk indicates that the confidence in this entry is less than in the others. The target node PA is underlined.

ABA	**PLDα**	**PLDδ**	**PC[1]**	**DAG**	**DAGK[2]**	**PA**
0	0	0	1	0	1	0
1	0	0	1	1	1	0
1	0	1	1	1	1	0
1	1	0	1	1	1	0*
1	1	1	1	1	1	1

Most likely rule: PA* = PLDα and PLDδ and PC and DAG and DAGK

Footnotes:

1. Phosphatidylcholine is a substrate and assumed to always be present.

2. DAGK is an enzyme, and there are no knockout experiments or regulators known for it. We assume it is always ON.

Supplementary Table S4. Truth table for AnionEM

Table produced from information in (16, 17, 25). The target node AnionEM is underlined.

ABI1	OST1	**SLAH3**	**SLAC1**	**QUAC1**	**AnionEM**
0	0	0	0	0	0
0	1	1	0	1	0
1	0	0	0	0	0
0	1	1	1	1	1
0	1	0	1	1	1
0	1	0	1	0	1

ABI1 inhibits SLAH3 (acts as the only regulator when assuming that the CPKs are present); OST1 is the only regulator of QUAC1. This truth table covers almost half of the state combinations of SLAC1, SLAH3 and QUAC1. There is no appropriate Boolean rule that perfectly fits the partial truth table. The Boolean rule with the best partial fit (namely, one error out of 6) for the above table is:

AnionEM* = SLAC1 or (SLAH3 and QUAC1).

Hence, our predicted Boolean rule is the same as the published rule.

Supplementary Table S5. Truth table for OST1

The recessive double knockout mutants of *abi1-abi2* (136); *abi1-hab1* (137); *hab1-pp2ca1* (138); and *abi1-pp2ca1* (138) show higher stomatal responsiveness to ABA. Since we know that all these PP2Cs inhibit OST1 (4,18,19,89), we can assign the effect of any PP2C double mutant as the ON state of OST1. This gives the following incomplete truth table:

**ABI1**	**ABI2**	**HAB1**	**PP2CA**	**OST1**
0	0	?	?	1
0	?	0	?	1
?	?	0	0	1
0	?	?	0	1

A parsimonious extension of this truth table where the ?'s are filled with all possible combinations of 0's and 1's yields the following Boolean rule:

OST1*= (not ABI1 and not HAB1) or (not PP2CA and not ABI2) or (not ABI1 and not ABI2) or (not HAB1 and not PP2CA) or (not HABI1 and not ABI2) or (not ABI1 and not PP2CA)

Please note that the parsimonious extension assumes that the recessive double knockout mutant of *abi2-hab1*; and *abi2-pp2ca1* will show higher stomatal responsiveness to ABA.

Supplementary Text S1. Inferred Boolean rules of the ABA network

Here we list all the Boolean rules inferred using causal logic inference for the ABA network. The rules that are different compared to previously published data (1) are marked in bold. The rules in italics are equivalent to the published rules in (1) after considering causal logic reduction.

8-nitro-cGMP = cGMP*

ABI1* = ROP11 and pHc and not RCARs and not ROS and not PA

ABI2* = ROP11 and not RCARs and not ROS

ADPRc* = 8-nitro-cGMP

AGB1* = GPA1

AGG3* = AGB1

Actin Reorganization* = SCAB1 and not AtRAC1 and PtdInsP3 and PtdInsP4 and ARP2/3 Complex

AnionEM* = (SLAH3 and QUAC1) or SLAC1

AtRAC1* = ABI1 or not ABA

CIS = InsP3 or cADPR or ABA or InsP6*

CPK3/21* = Ca2+ or CPK3/21

Ca2+* = (CIS or CaIM) and not Ca2+ ATPase

Ca2+ ATPase* = Ca2+

CaIM* = Actin Reorganization or (NtSyp121 and GHR1 and MRP5) or not ABH1 or not ERA1 or OST1

DAG* = PLC and PtdIns(4,5)P2

Depolarization* = (Ca2+ or KEV or AnionEM) and (not K+ Efflux or not H+ ATPase)

GHR1* = not ABI2 and ROS

GPA1* = S1P or not GCR1

H+ ATPase* = not Ca2+ and not pHc and not ROS

H2O Efflux* = PIP2;1 and K+ Efflux and AnionEM and not Malate

HAB1* = not RCARs and not ROS

InsP3* = PtdIns(4,5)P2 and PLC

InsP6* = InsP3

K+ Efflux* = KOUT and KEV

KEV* = Ca2+

KOUT* = Depolarization and not ROS and pHc and not NO

MPK* = Ca2+ or MPK

Malate* = PEPC and not ABA and not AnionEM

Microtubule depolymerization = TCTP or ROS or ABA or Microtubule depolymerization*

NIA1/2* = ROS

NO* = NADPH and Nitrite and NIA1/2

NOGC1* = NO

NtSyp121* = ABA

OST1* = (not ABI1 and not HAB1) or (not PP2CA and not ABI2) or (not ABI1 and not ABI2) or (not HAB1 and not PP2CA) or (not HABI1 and not ABI2) or (not ABI1 and not PP2CA)

PA* = DAG and DAGK and PC and PLDa and PLDdel

PEPC* = not ABA

PI3P5K* = ABA
PIP2;1* = OST1
PLC* = Ca2+
PLDa* = Ca2+ and GPA1
PLDdel* = NO or (ROS and GAPC1/2)
PP2CA* = not RCARs
PtdIns(3,5)P2* = PI3P5K
PtdIns(4,5)P2* = PtdInsP4
QUAC1* = OST1 and Ca2+
RBOH = PA and pHc and OST1 and GPA1 and not ABI1*
RCARs* = ABA
ROP10* = ERA1
ROP11* = GEF1/4/10
ROS = RBOH and NADPH and PtdInsP3 and RCN1*
S1P* = Sph and SPHK1/2 and not SPP1
SLAC1* = (CPK23 or CKP6 or CPK3/21) and pHc and GHR1 and MRP5 and OST1 and not ABI1 and not ABI2 and not PP2CA and MPK
SLAH3* = (CPK23 or CKP6 or CPK3/21) and not ABI1
SPHK1/2* = ABA or PA
Stomatal closure* = cADPR and NtSyp121 and S1P and CIS and not H^+ ATPase and Microtubule Depolymerization and H_2O efflux
TCTP* = Ca2+
V-ATPase* = Ca2+
V-Ppase* = PtdIns(3,5)P2
Vacuolar acidification* = V-Ppase and V-ATPase
cADPR* = ADPRc and NAD+
cGMP* = NOGC1 and GTP
pHc* = Ca2+ and OST1 and Vacuolar acidification and not ABI1 and not ABI2

Supplementary Text S2. Differences between the inferred Boolean functions and the published Boolean functions in the ABA network

There are 13 nodes in the ABA network that have different Boolean rules obtained from the inference method as compared to published rules (1). Here, we divide these 13 cases in three groups. The first group contains the nodes for which both the inferred rules and the published rules have the same regulators, but they differ in the Boolean operators used. The second group contains the nodes for which the inferred rule has fewer regulators compared to the published rule. And the third group contains the nodes for which the inferred rule has more regulators compared to the published rule. For each node listed here, the inferred rule is in black, bold and italic font. The text under the rules in italics explains the rationale for the inferred rule. The published rule from (1) is below the explanation in black, bold font followed by

text that is a quote from Text S2 of (1) relevant for the reasoning for the published rule and to the discrepancy. The citation numbers in the quote refer to the references in this file.

Group 1. The published and inferred functions contain the same regulators but there are differences in Boolean operators. The inferred function is equally or more consistent with the experimental information than the published function.

Actin Reorganization* = PtdInsP3 and PtdInsP4 and not AtRAC1 and ARP2/3 Complex and SCAB1

During the inference process the ARP complex is marked as necessary for Actin Reorganization; PtdInsP3 and PtdInsP4 are necessary; AtRAC1 is a sufficient inhibitor for Actin Reorganization. If SCAB1 is assumed to be present in all these experiments (1), *we get the resulting rule. There are no experiments that address whether PtdInsP3 and PtdInsP4 are redundant (as assumed in the published function) or not (as in the inferred function).*

Actin Reorganization*= (PtdInsP4 or PtdInsP3) and not AtRAC1 and ARP Complex and SCAB1

“Expression of a dominant-positive mutant of AtRAC1 inhibits ABA-induced actin reorganization whereas expression of a dominant-negative mutant of AtRAC1 promotes actin reorganization in the absence of ABA (49). Inhibition of AtRAC1 is necessary for actin reorganization in response to ABA. ARP2 knockouts did not exhibit actin reorganization during ABA signaling. The *arp2* phenotype was rescued upon application of an actin depolymerizing agent which illustrates that ARP2 is a positive regulator of the actin reorganization process (27). In this particular case, the behavior of one subunit is assumed to describe the behavior of the protein complex (27). In Arabidopsis, SCAB1 encodes a plant specific actin binding protein. The *scab1* loss-of-function mutant shows a delayed ABA response that is associated with delayed actin reorganization (100). Both PtdInsP4 and PtdInsP3 are implicated as positive regulators of actin reorganization in response to ABA (90).”

ABI1* = not PA and not RCARs and ROP11 and not ROS and pHc

According to causal logic inference, pHc is necessary for ABI1, and each of RCARs, ROS, PA is a sufficient inhibitor of ABI1. The existing information on the effect of ROP11 on ABI1 (93) *does not lead to a strong logic implication. To achieve compatibility, the Boolean “and” operator is extended to ROP11.*

ABI1*=not PA and (not RCARs or ROP11) and not ROS and pH_c

“RCARs inhibit ABI1 through physical binding (6). ROP11 interacts with ABI1 and protects it from inhibition by RCARs (93). Upon binding PA inhibits phosphatase activity of ABI1 (80). PA also tethers ABI1 to the plasma membrane, which in turn negatively regulates ABI1 function (79). ROS inhibit ABI1 activity (97). Cytosolic pH increase activates enzyme activity of ABI1 (84).”

ABI2* = not RCARs and ROP11 and not ROS

Each of RCARs and ROS is a sufficient inhibitor of ABI2. As in the function of ABI1, the Boolean "and" operator is extended to ROP11.

ABI2*= (not RCARS or ROP11) and not ROS

"ROP11 physically interacts with ABI2 and promotes its phosphatase activity (58). RCARs inhibit phosphatase activity of ABI2 by physical binding (6). ABI2 has been shown to be negatively regulated by ROS (98); we assume that the absence or low level of ROS is a necessary condition for ABI2 activity. In addition to this requirement, we assume that in order for ABI2 to be active, its positive regulator ROP11 must be active or its other negative regulators, RCARs, must be off."

KOUT = not NO and not ROS and pHc and Depolarization

During inference, we marked NO and ROS as sufficient inhibitors of KOUT, pH_c as sufficient and Depolarization as necessary for KOUT. These logic implications are incompatible. The inferred function uses the assumption that the experiment addressing pHc involved the absence of ROS and NO and the presence of membrane depolarization. The published function involves the knowledge that is equivalent with assuming that the experiments addressing the role of pHc, ROS or NO involved membrane depolarization.

KOUT*= (not NO or not ROS or pH_c) and Depolarization

"Membrane depolarization drives K^+ efflux from the guard cell. Outwardly rectifying K^+ channels are activated by cytosolic pH increase (82) and inhibited by ROS (95) and nitric oxide (70). K^+ efflux through outwardly rectifying K^+ channels requires membrane depolarization; thus we use an "and" function between "Depolarization" and other indicated positive or negative regulators of KOUT. In the absence of documented synergy, we use an "or" function between NO, ROS and pH_c."

PA* = PC and PLDδ and PLDα and DAG and DAGK

The regulators of PA have logic implications with a lower confidence; hence we construct an incomplete truth table, see Table S5. The inferred function reproduces all the entries of the truth table. The published function contains the judgement call that activity of any of the three reactions that produce PA is sufficient for above-threshold PA concentration. This function does not reproduce three entries of the truth table (it yields 1 instead of 0).

PA*= PC and (PLDδ or PLDα) or DAG and DAGK

"PC is the substrate needed by PLDα or PLDδ for PA production. DAG, a product of PLC, can be converted into PA by DAGK-mediated phosphorylation (52,113)."

pH_c* = OST1 and not ABI2 and not ABI1 and $Ca^{2+}{}_c$ and Vacuolar acidification

During inference, we marked ABI1 and ABI2 as sufficient inhibitors of pH_c and OST1, Vacuolar Acidification, and $Ca^{2+}{}_c$ as necessary for pH_c increase based on experiments done in the presence of ABA.

The published function also uses specific evidence from external calcium induced closure to group regulators whose effect can be overcome in this process.

pH_c*= (OST1 and not ABI2 and not ABI1 or $Ca^{2+}{}_c$) and Vacuolar acidification

"Guard cells of *abi1-1* (dominant negative), *abi2-1* (dominant negative), and *ost1-2* loss of function mutants show impaired cytosolic alkalization in response to ABA (31). Exogenous calcium application is assumed to increase $Ca^{2+}{}_c$ concentration and can induce cytosolic alkalization in *ost1-2* (loss-of-function), *abi1-1* (dominant negative), *and abi2-1* (dominant negative) mutants (31), indicating that $Ca^{2+}{}_c$-triggered pH_c increase in guard cells does not require functional OST1 or the inactivation of ABI1 or ABI2; hence the OR relationship between $Ca^{2}{}_{\mathbf{c}}$ and these three proteins. External application of a calcium chelator, EGTA, reduces ABA-induced cytosolic alkalization (pH_c increase) (105), indicating that $Ca^{2+}{}_c$ is a positive regulator in ABA-induced cytosolic alkalization. Vacuolar acidification is a necessary condition for maintenance of ABA induced cytosolic alkalization state in guard cells (8). Thus, we use an "and" function between Vacuolar acidification and other indicated positive and negative regulators of pH_c increase."

SLAH3* = (CKP6 or CPK23 or CPK3/21) and not ABI1

The causal logic of regulatory relationships indicates that any of the CPKs are sufficient for SLAH3 activity and that ABI1 is a sufficient inhibitor of SLAH3. The evidence for the effect of ABI1 on SLAH3 is stronger, hence we use the dominant regulators method with ABI1 as a dominant regulator. The published function uses a relationship between ABA and CPK3/21 that was not in the data source used for inference.

SLAH3*= (CPK6 or CPK23) and CPK3/21 and not ABI1

"All listed CPKs activate SLAH3 by physical interaction (16,37). All indicated CPKs have an independent positive effect on SLAH3. ABI1 inhibits CPK21-mediated activation of SLAH3 in oocytes indicating that ABI1 is a negative regulator of SLAH3 (16). Since CPK6 and CPK23 are weakly dependent on ABA (130), we implement the dependence of SLAH3 activation on ABA (16,37) by assuming that only the simultaneous activity of CPK6 and CPK3/21, or CPK23 and CPK3/21, is sufficient for SLAH3 activation."

Group 2. The manually constructed regulatory function has more regulators. The inferred function is equally consistent with the experimental information as the published function.

PP2CA* = not RCARs

No regulatory relationship from ROS to PP2CA is in the data source used for inference. This relationship was assumed in the published version.

PP2CA*= not RCARS and not ROS

"In an *in vitro* study, it has been shown that soluble ABA receptors (RCARs, alternatively known as PYR/PYLs) PYR1, PYL1, PYL2, PYL4, PYL5, PYL6, PYL8 inhibit the phosphatase activity of PP2CA in the presence of ABA (92). ROS-mediated inhibition has been reported for three PP2C-type protein phosphatases that play negative regulatory roles in guard cell ABA signaling: ABI1, ABI2 and HAB1 (97–99). We assume that ROS would similarly inhibit the phosphatase activity of PP2CA."

Vacuolar acidification* = V-Ppase and V-ATPase

We marked both V-ATPase and V-Ppase as necessary for vacuolar acidification. The self-regulatory relationship of Vacuolar Acidification assumed in the published function is based on a context other than ABA-induced closure. We note that our recent work provides an alternative to making this assumption (131).

Vacuolar Acidification*= V-Ppase or V-ATPase or Vacuolar Acidification

"In yeast, the vacuolar proton ATPase (V-ATPase) proton pump plays an important role in vacuolar acidification (102,103). The proton pumping vacuolar pyrophosphatase (V-Ppase) uses the energy of PP_i hydrolysis to acidify the vacuole (132). An Arabidopsis V-Ppase loss-of-function mutant, *vhp1,* shows delayed vacuolar acidification and slower stomatal closure in response to ABA (8,133). An Arabidopsis double knockout mutant of V-ATPase, *vha1 vha2,* exhibits a vacuolar pH of 6.4 rather than 5.9 (134). The double knockout mutant of the V-ATPase also shows delayed stomatal closure in response to ABA (8). We used an "or" function between V-Ppase and V-ATPase as the V-Ppase and V-ATPase play independent roles in vacuolar acidification (8). We assume that the vacuolar acidification state is sustained for a longer period and implement this assumption as a positive self-regulation. This assumption is necessary in order to allow the possibility of closure in response to internal closure signals (e.g. supply of S1P or Ca^{2+} (135))"

KEV* = Ca^{2+}_c

The published function contained a mistake in citing Vacuolar identification as a regulator of the K+ channel KEV. The experimental observation in Figure 6B of reference (34) indicates that the conductance of this channel decreases five-fold as cytosolic pH increases from 6.5 to 8. During ABA induced closure pH_c changes by about 0.3 starting from the value of 7.3 (86); the conductance of the KEV channel changes very little in this range.

KEV*: Vacuolar Acidification or Ca^{2+}_c

Calcium induces K^+ release through K^+-permeable channels in the tonoplast (34). Vacuolar acidification also induces K^+ efflux from the vacuole (34).

Group 3. The inferred regulatory function has more regulators. The inferred function is equally consistent with the experimental information as the published function.

SLAC1* = (CKP6 or CPK23 or CPK3/21) and MPK and OST1 and GHR1 and not ABI1 and not PP2CA and not ABI2 and pHc and MRP5

MRP5 is necessary for Ca^{2+}_c activation of SLAC1 (123)*. Because there isn't a path or subgraph that can mediate this effect, MRP5 appears as necessary regulator in the inferred rule. MRP5 is not present as regulator in the published rule; instead, it is assumed to be a regulator of CaIM.*

SLAC1*= (CPK6 or CPK23 or CPK3/21) and MPK9/12 and OST1 and GHR1 and not ABI1 and not PP2CA and not ABI2 and pH_c

CaIM* = Actin Reorganization or (NtSyp121 and GHR1 and MRP5) or not ABH1 or not ERA1 or OST1
The inferred rule has the regulator OST1 which is not present in the published rule. The inference process finds that OST1 is sufficient for CaIM. The network has no sufficient path or subgraph from OST1 to CaIM (the path OST1→RBOH→ROS→GHR1→CaIM is not sufficient), hence we add this as an edge.
CaIM*= Actin Reorganization or (NtSyp121 and GHR1 and MRP5) or not ABH1 or not ERA1

Closure* = Microtubule Depolymerization and H_2O efflux and cADPR and NtSyp121 and S1P and CIS and not H+ ATPase
The list of regulatory relationships in the inference process has multiple regulators that are found to be necessary for closure. Some of the regulators are reduced if a necessary path or subgraph to closure already exists but several regulators are not reduced and hence appear in the Boolean rule for stomatal closure. The published function is based on the biological knowledge that two independent processes are responsible for the shape and volume changes of the guard cells needed for stomatal closure.
Closure*= Microtubule Depolymerization and H_2O efflux
"Microtubule depolymerization and H_2O efflux are both needed for stomatal closure (66)."

Supplementary Table S6. Derived logic observations for the inference of the EMT network

Each row lists a logic observation denoted by a regulator node, target node, the corresponding logic implication (s, n, si, ni, sn, or sni) and a Boolean marker (Y/N) for whether the edge is expected to be direct or indirect. Highlighted in blue are all the indirect edges. These are reduced by the code in the inference process. Each of them has a mediator between the regulator and the target, and for each, regulator → mediator as well as mediator → target inference information exists in this list. Highlighted in green are all the cases in which we infer the mediator node. The mediators for the three cases are: CDC42, CD44, and betaTrCP, respectively. Highlighted in pink is the case where an incomplete path from the regulator (TCF/LEF) to the target (SHH) is known to be transduced via a potential mediator node (GLI) helping us infer an edge between the regulator and mediator thus completing the indirect observation of regulator (TCF/LEF) effect on target (SHH). Highlighted in sea green is an example of the co-pointing theorem potentially being applied to this network. Assuming the presence of betacatenin_memb, RAS is sufficient inhibitory for E-cadherin but TWIST1 is necessary inhibitory for E-cadherin. Sufficient inhibitory and

necessary inhibitory are incompatible logic implications hence extending the co-pointing theorem, RAS must be sufficient for TWIST1. The rest of the list helps us infer that RAS is sufficient for TWIST1, and hence the co-pointing theorem application here just acts as supporting evidence.

**Regulator**	**Target**	**Logic implication**	**Is edge direct?**
ILK	AKT	s	Y
PI3K	AKT	s	Y
GSK3beta	Dest_compl	n	Y
AXIN2	Dest_compl	n	Y
betacatenin_nuc	Dest_compl	n	Y
GSK3beta	Dest_compl	s	Y
Dest_compl	Dest_compl	n	Y
AXIN2	AXIN2	s	Y
TCF/LEF	AXIN2	s	Y
E-cadherin	betacatenin_memb	n	Y
betacatenin_nuc	betacatenin_memb	si	Y
Dest_compl	betacatenin_nuc	si	Y
betacatenin_memb	betacatenin_nuc	si	Y
SUFU	betacatenin_nuc	ni	Y
E-cadherin	betacatenin_nuc	ni	Y
CHD1L	CDC42	s	Y
ERK	c-fos	sn	Y
HGF	cMet	s	Y
NOTCH_ic	Csl	sn	Y
NFkB	Csn	sn	Y
RAS	DELTA	sn	Y
Frizzled	DSH	sn	Y
betacatenin_memb	E-cadherin	n	Y
SNAI1	E-cadherin	ni	Y
HEY1	E-cadherin	ni	Y
ZEB1	E-cadherin	ni	Y

ZEB2	E-cadherin	ni	Y
FOXC2	E-cadherin	ni	Y
TWIST1	E-cadherin	ni	Y
SNAI2	E-cadherin	ni	Y
EGF	EGFR	sn	Y
c-fos	EGR1	sn	Y
E-cadherin	EMT	ni	Y
EMT	EMT	s	Y
MEK	ERK	sn	Y
FGF	FGFR	sn	Y
Goosecoid	FOXC2	s	Y
SNAI1	FOXC2	s	Y
TWIST1	FOXC2	s	Y
Wnt	Frizzled	sn	Y
SMO	FUS	sn	Y
TCF/LEF	GLI	s	Y
SUFU	GLI	ni	Y
DSH	GSK3beta	si	Y
AKT	GSK3beta	si	Y
Csn	GSK3beta	ni	Y
ERK	GSK3beta	ni	Y
Dest_compl	GSK3beta	ni	Y
Csl	HEY1	s	Y
SMAD	HEY1	s	Y
Hypoxia	HIF1a	sn	Y
IGF1	IGF1R	sn	Y
AKT	IKKa	sn	Y
SMAD	ILK	sn	Y
TCF/LEF	Jagged	s	Y
SMAD	Jagged	s	Y
STAT	LIV1	sn	Y
HIF1a	LOXL23	sn	Y
RAF	MEK	s	Y

RKIP	MEK	ni	Y
SNAI1	miR200	si	Y
ZEB1	miR200	si	Y
ZEB2	miR200	si	Y
IKKa	NFKb	sn	Y
DELTA	NOTCH	s	Y
NOTCH	NOTCH_ic	sn	Y
SHH	Patched	sni	Y
PDGF	PDGFR	sn	Y
RAS	PI3K	sn	Y
RAS	RAF	sn	Y
SOS/GRB2	RAS	s	Y
SRC	RAS	s	Y
GSK3beta	RAS	ni	Y
TCF/LEF	RAS	s	Y
ERK	RKIP	ni	Y
SNAI1	RKIP	ni	Y
SMAD	SHH	s	Y
ERK	SNAI2	s	Y
betacatenin_nuc	SNAI2	s	Y
SNAI2	SNAI2	s	Y
TWIST1	SNAI2	s	Y
ERK	SMAD	s	Y
TGFbR	SMAD	n	Y
ZEB1	SMAD	n	Y
ZEB2	SMAD	si	Y
Patched	SMO	sn	Y
GLI	SNAI1	s	Y
LOXL23	SNAI1	s	Y
SMAD	SNAI1	s	Y
LIV1	SNAI1	s	Y
PAK1	SNAI1	s	Y
Csl	SNAI1	s	Y

EGR1	SNAI1	s	Y
Goosecoid	SNAI1	s	Y
GSK3beta	SNAI1	ni	Y
PDGFR	SOS/GRB2	s	Y
cMet	SOS/GRB2	s	Y
Jagged	NOTCH	s	Y
TGFbR	SOS/GRB2	s	Y
FGFR	SOS/GRB2	s	Y
IGF1R	SOS/GRB2	s	Y
EGFR	SOS/GRB2	s	Y
ERK	SOS/GRB2	si	Y
SRC	STAT	sn	Y
FUS	SUFU	sni	Y
betacatenin_nuc	TCF/LEF	sn	Y
Goosecoid	TGFb	s	Y
SNAI1	TGFb	s	Y
TWIST1	TGFb	s	Y
GLI1	TGFb	s	Y
TGFb	TGFbR	sn	Y
NFkB	TWIST1	s	Y
HIF1	TWIST1	s	Y
TCF/LEF	TWIST1	s	Y
Goosecoid	TWIST1	s	Y
SNAI1	TWIST1	s	Y
GLI	Wnt	sn	Y
HIF1a	ZEB1	s	Y
SNAI1	ZEB1	s	Y
Goosecoid	ZEB1	s	Y
miR200	ZEB1	si	Y
HIF1a	ZEB2	s	Y
SNAI1	ZEB2	s	Y
Goosecoid	ZEB2	s	Y
miR200	ZEB2	si	Y

PI3K	GSK3beta	si	N
TGFbR	PAK1	s	N
TCF/LEF	SNAI1	s	N
TCF/LEF	Wnt	s	N
DSH	RAS	s	N
AKT	RAS	s	N
SMAD	NOTCH	s	N
TCF/LEF	NOTCH	s	N
RAF	ERK	s	N
IKKa	TWIST1	s	N
DELTA	NOTCH_ic	s	N
RAS	AKT	s	N
Jagged	NOTCH_ic	s	N
TGFbR	PAK1	s	N
TCF/LEF	cMET	s	N
Csn	SNAI1	ni	N
TCF/LEF	SHH	s	N
RAS	E-cadherin	si	N
TWIST1	E-cadherin	ni	N
RAS	*TWIST1*	*s*	

Supplementary Text S3. Inferred Boolean functions for the EMT network

Regulators of Dest_compl are incompatible. Rules as per the two templates are:

1. (GSK3beta) and Dest_compl and betacatenin_nuc and AXIN2

2. GSK3beta or (Dest_compl and betacatenin_nuc and AXIN2)

Csl* = NOTCH_ic

TGFbR* = TGFb

Csn* = NFkB

c-fos* = ERK

GLI* = TCF/LEF or not SUFU

miR200* = not SNAI1 and not ZEB1 and not ZEB2

Regulators of SMAD are incompatible. Rules as per the two templates are:

1. (ERK) and TGFbR and not ZEB2 and ZEB1

2. ERK or (TGFbR and not ZEB2 and ZEB1)

EGFR* = EGF

ILK* = SMAD

AKT* = ILK or PI3K

SMO* = Patched

SNAI2* = SNAI2 or TWIST1 or betacatenin_nuc or ERK

Patched* = not SHH

PDGF is a source node

IKKa* = AKT

HIF1a* = Hypoxia

STAT* = SRC

NOTCH_ic* = NOTCH

EGR1* = c-fos

Frizzled* = Wnt

EGF is a source node

ERK* = MEK

RAF* = RAS

betaTrCP* = not Csn

NOTCH* = Jagged or DELTA

AXIN2* = TCF/LEF or AXIN2

Regulators of GSK3beta are incompatible. Rules as per the two templates are:

1. (not DSH and not AKT) or not Dest_compl or not Csn or not ERK

2. not DSH and not AKT and (not Dest_compl or not Csn or not ERK)

TWIST1* = HIF1 or NFkB or TCF/LEF or Goosecoid or SNAI1

cMet* = HGF or CD44

Regulators of E-cadherin are incompatible. Rules as per the two templates are:

1. (betacatenin_memb) or not HEY1 or not TWIST1 or not SNAI2 or not SNAI1 or not ZEB1 or not FOXC2 or not ZEB2

2. betacatenin_memb and (not HEY1 or not TWIST1 or not SNAI2 or not SNAI1 or not ZEB1 or not FOXC2 or not ZEB2)

Regulators of ZEB2 are incompatible. Rules as per the two templates are:

1. (not miR200) or SNAI1 or Goosecoid or HIF1a

2. not miR200 and (SNAI1 or Goosecoid or HIF1a)

Hypoxia is a source node

RAS* = SRC or SOS/GRB2 or TCF/LEF or not GSK3beta

Wnt* = GLI

FGF is a source node

HEY1* = Csl or SMAD

FGFR* = FGF

SRC is a source node

IGF1R* = IGF1

IGF1 is a source node

TGFb* = TWIST1 or GLI1 or SNAI1 or Goosecoid

LIV1* = STAT

PI3K* = RAS

Goosecoid is a source node

CD44* = TCF/LEF

NFkB is a source node

betacatenin_memb* = not betacatenin_nuc and E-cadherin

TCF/LEF* = betacatenin_nuc

DSH* = Frizzled

SUFU* = not FUS

HGF is a source node

LOXL23* = HIF1a

SNAI1* = LOXL23 or GLI or Goosecoid or LIV1 or Csl or EGR1 or not betaTrCP or not GSK3beta or SMAD or PAK1

Regulators of ZEB1 are incompatible. Rules as per the two templates are:

1. (not miR200) or SNAI1 or Goosecoid or HIF1a

2. not miR200 and (SNAI1 or Goosecoid or HIF1a)

CHD1L is a source node

GLI1 is a source node

RKIP* = not SNAI1 or not ERK

EMT* = EMT or not E-cadherin

HIF1 is a source node

SHH* = GLI or SMAD

PDGFR* = PDGF

MEK* = RAF or not RKIP

PAK1* = CDC42

FUS* = SMO

NFKb* = IKKa

Regulators of SOS/GRB2 are incompatible. Rules as per the two templates are:

1. (not ERK) or IGF1R or cMet or EGFR or TGFbR or PDGFR or FGFR

2. not ERK and (IGF1R or cMet or EGFR or TGFbR or PDGFR or FGFR)

DELTA* = RAS

Jagged* = TCF/LEF or SMAD

Regulators of betacatenin_nuc are incompatible. Rules as per the two templates are:

1. (not E-cadherin or not SUFU) and not Dest_compl and not betacatenin_memb

2. not E-cadherin or not SUFU or (not Dest_compl and not betacatenin_memb)

FOXC2* = TWIST1 or SNAI1 or Goosecoid

CDC42* = TGFbR or CHD1L

Supplementary Table S7. Reduced input logic observations for modified EMT network inference

Each row lists a logic observation denoted by a regulator node, target node, the corresponding logic implication (s, n, si, ni, sn, or sni) and a Boolean marker (Y/N) for whether the edge is expected to be direct or indirect. This table contains 118 rows that were randomly chosen from Table S8. The contents of this table are used as input to generate the output Boolean rules presented in Text S11.

**Regulator**	**Target**	**Logic implication**	**Is edge direct?**
ILK	AKT	s	Y
PI3K	AKT	s	Y
AXIN2	Dest_compl	n	Y
betacatenin_nuc	Dest_compl	n	Y
GSK3beta	Dest_compl	s	Y
Dest_compl	Dest_compl	n	Y
TCF/LEF	AXIN2	s	Y

E-cadherin	betacatenin_memb	n	Y
Dest_compl	betacatenin_nuc	si	Y
betacatenin_memb	betacatenin_nuc	si	Y
SUFU	betacatenin_nuc	ni	Y
E-cadherin	betacatenin_nuc	ni	Y
CHD1L	CDC42	s	Y
ERK	c-fos	sn	Y
HGF	cMet	s	Y
NOTCH_ic	Csl	sn	Y
NFkB	Csn	sn	Y
RAS	DELTA	sn	Y
Frizzled	DSH	sn	Y
betacatenin_memb	E-cadherin	n	Y
SNAI1	E-cadherin	ni	Y
HEY1	E-cadherin	ni	Y
ZEB1	E-cadherin	ni	Y
FOXC2	E-cadherin	ni	Y
TWIST1	E-cadherin	ni	Y
SNAI2	E-cadherin	ni	Y
EGF	EGFR	sn	Y
E-cadherin	EMT	ni	Y
EMT	EMT	s	Y
MEK	ERK	sn	Y
FGF	FGFR	sn	Y
Goosecoid	FOXC2	s	Y
SNAI1	FOXC2	s	Y
Wnt	Frizzled	sn	Y
SMO	FUS	sn	Y
TCF/LEF	GLI	s	Y
SUFU	GLI	ni	Y
DSH	GSK3beta	si	Y
AKT	GSK3beta	si	Y
ERK	GSK3beta	ni	Y

Dest_compl	GSK3beta	ni	Y
Csl	HEY1	s	Y
Hypoxia	HIF1a	sn	Y
IGF1	IGF1R	sn	Y
AKT	IKKa	sn	Y
SMAD	ILK	sn	Y
TCF/LEF	Jagged	s	Y
SMAD	Jagged	s	Y
HIF1a	LOXL23	sn	Y
RAF	MEK	s	Y
RKIP	MEK	ni	Y
ZEB1	miR200	si	Y
ZEB2	miR200	si	Y
IKKa	NFKb	sn	Y
DELTA	NOTCH	s	Y
NOTCH	NOTCH_ic	sn	Y
SHH	Patched	sni	Y
PDGF	PDGFR	sn	Y
RAS	PI3K	sn	Y
RAS	RAF	sn	Y
SOS/GRB2	RAS	s	Y
SRC	RAS	s	Y
TCF/LEF	RAS	s	Y
ERK	RKIP	ni	Y
SNAI1	RKIP	ni	Y
SMAD	SHH	s	Y
ERK	SNAI2	s	Y
SNAI2	SNAI2	s	Y
TWIST1	SNAI2	s	Y
ERK	SMAD	s	Y
ZEB1	SMAD	n	Y
ZEB2	SMAD	si	Y
Patched	SMO	sn	Y

GLI	SNAI1	s	Y
LOXL23	SNAI1	s	Y
SMAD	SNAI1	s	Y
PAK1	SNAI1	s	Y
Csl	SNAI1	s	Y
EGR1	SNAI1	s	Y
Goosecoid	SNAI1	s	Y
GSK3beta	SNAI1	ni	Y
PDGFR	SOS/GRB2	s	Y
cMet	SOS/GRB2	s	Y
TGFbR	SOS/GRB2	s	Y
FGFR	SOS/GRB2	s	Y
EGFR	SOS/GRB2	s	Y
ERK	SOS/GRB2	si	Y
SRC	STAT	sn	Y
SNAI1	TGFb	s	Y
TWIST1	TGFb	s	Y
GLI1	TGFb	s	Y
NFkB	TWIST1	s	Y
HIF1	TWIST1	s	Y
TCF/LEF	TWIST1	s	Y
SNAI1	TWIST1	s	Y
HIF1a	ZEB1	s	Y
SNAI1	ZEB1	s	Y
Goosecoid	ZEB1	s	Y
miR200	ZEB1	si	Y
HIF1a	ZEB2	s	Y
Goosecoid	ZEB2	s	Y
miR200	ZEB2	si	Y
PI3K	GSK3beta	si	N
TGFbR	PAK1	s	N
AKT	RAS	s	N
SMAD	NOTCH	s	N

RAF	ERK	s	N
IKKa	TWIST1	s	N
RAS	AKT	s	N
Jagged	NOTCH_ic	s	N
TGFbR	PAK1	s	N
TCF/LEF	cMET	s	N
Csn	SNAI1	ni	N
TCF/LEF	SHH	s	N
RAS	E-cadherin	si	N
TWIST1	E-cadherin	ni	N
RAS	*TWIST1*	*s*	

Supplementary Text S5. Inferred Boolean functions for the modified EMT network

This text presents the inferred Boolean functions using our inference method for the reduced input information as provided in Table S10. The resulting Boolean functions are identical to the expected set of functions (see Text S9) except in the case of 18 nodes that are highlighted below in bold. The majority of cases of discrepancy (16) consist of missing a regulator; in 6 of these cases this omission creates a source node. There is one function (that of SNAI2) from which 2 regulators are missing. Indeed, in all of these cases the regulator-target relationship was missing from the input information. Finally, there is one function (that of RAS) in which one regulator is replaced (it contains “or AKT” instead of “or not GSK3beta”.

The regulators of Dest_compl are incompatible. The rules as per the two templates are:

1. (GSK3beta) and Dest_compl and betacatenin_nuc and AXIN2
2. GSK3beta or (Dest_compl and betacatenin_nuc and AXIN2)

Csl* = NOTCH_ic

TGFbR is a source node

Csn* = NFkB

c-fos* = ERK

GLI* = TCF/LEF or not SUFU

miR200* = not ZEB1 and not ZEB2

Regulators of SMAD are incompatible. The rules as per the two templates are:

1. (ERK) and not ZEB2 and ZEB1

2. ERK or (not ZEB2 and ZEB1)

EGFR* = EGF

ILK* = SMAD

AKT* = ILK or PI3K

SMO* = Patched

SNAI2* = SNAI2 or TWIST1

Patched* = not SHH

PDGF is a source node

IKKa* = AKT

RKIP* = not SNAI1 or not ERK

STAT* = SRC

NOTCH_ic* = NOTCH

NOTCH* = Jagged or DELTA

Frizzled* = Wnt

EGF is a source node

ERK* = MEK

RAF* = RAS

betaTrCP is a source node

CHD1L is a source node

The regulators of GSK3beta are incompatible. The rules as per the two templates are:

1. (not PI3K and not DSH) or not Dest_compl or not ERK

2. not PI3K and not DSH and (not Dest_compl or not ERK)

TWIST1* = IKKa or HIF1 or NFkB or TCF/LEF or SNAI1

The regulators of E-cadherin are incompatible. The rules as per the two templates are:

1. (betacatenin_memb) or not TWIST1 or not SNAI2 or not SNAI1 or not ZEB1 or not FOXC2 or not HEY1

2. betacatenin_memb and (not TWIST1 or not SNAI2 or not SNAI1 or not ZEB1 or not FOXC2 or not HEY1)

HIF1a* = Hypoxia

Hypoxia is a source node

RAS* = SRC or SOS/GRB2 or TCF/LEF or AKT

Wnt is a source node

FGF is a source node

FGFR* = FGF

The regulators of ZEB2 are incompatible. The rules as per the two templates are:

1. (Goosecoid or HIF1a) and not miR200

2. Goosecoid or HIF1a or (not miR200)

SRC is a source node

IGF1R* = IGF1

IGF1 is a source node

EGR1 is a source node

PI3K* = RAS

Goosecoid is a source node

CD44* = TCF/LEF

NFkB is a source node

TCF/LEF is a source node

TGFb* = TWIST1 or SNAI1 or GLI1

DSH* = Frizzled

SUFU is a source node

HGF is a source node

LOXL23* = HIF1a

SNAI1* = LOXL23 or GLI or not GSK3beta or Csl or EGR1 or not betaTrCP or SMAD or PAK1

The regulators of ZEB1 are incompatible. The rules as per the two templates are:

1. (not miR200) or SNAI1 or Goosecoid

2. not miR200 and (SNAI1 or Goosecoid)

GLI1 is a source node

EMT* = EMT or not E-cadherin

HIF1 is a source node

SHH* = GLI or SMAD

PDGFR* = PDGF

MEK* = RAF or not RKIP

FUS* = SMO

NFKb* = IKKa

The regulators of SOS/GRB2 are incompatible. The rules as per the two templates are:

1. (not ERK) or TGFbR or cMet or EGFR or PDGFR or FGFR

2. not ERK and (TGFbR or cMet or EGFR or PDGFR or FGFR)

DELTA* = RAS

Jagged* = TCF/LEF or SMAD

The regulators of betacatenin_nuc are incompatible. The rules as per the two templates are:

1. (not E-cadherin or not SUFU) and not Dest_compl and not betacatenin_memb

2. not E-cadherin or not SUFU or (not Dest_compl and not betacatenin_memb)

FOXC2* = SNAI1 or Goosecoid

CDC42* = TGFbR or CHD1L

Bibliography

1. Albert R, Acharya BR, Jeon BW, Zañudo JG, Zhu M, Osman K, et al. A new discrete dynamic model of ABA-induced stomatal closure predicts key feedback loops. PLoS biology. 2017;15(9):e2003451.

2. Joudoi T, Shichiri Y, Kamizono N, Akaike T, Sawa T, Yoshitake J, et al. Nitrated cyclic GMP modulates guard cell signaling in Arabidopsis. The Plant Cell. 2013;25(2):558–571.

3. Park SY, Fung P, Nishimura N, Jensen DR, Fujii H, Zhao Y, et al. Abscisic acid inhibits type 2C protein phosphatases via the PYR/PYL family of START proteins. Science. 2009/05/02 ed. 2009 May 22;324(5930):1068–71.

4. Nishimura N, Sarkeshik A, Nito K, Park S-Y, Wang A, Carvalho PC, et al. PYR/PYL/RCAR family members are major in-vivo ABI1 protein phosphatase 2C-interacting proteins in Arabidopsis. The Plant Journal. 2010;61(2):290–299.

5. Gonzalez-Guzman M, Pizzio GA, Antoni R, Vera-Sirera F, Merilo E, Bassel GW, et al. Arabidopsis PYR/PYL/RCAR receptors play a major role in quantitative regulation of stomatal aperture and transcriptional response to abscisic acid. The Plant Cell. 2012;24(6):2483–2496.

6. Ma Y, Szostkiewicz I, Korte A, Moes D, Yang Y, Christmann A, et al. Regulators of PP2C phosphatase activity function as abscisic acid sensors. Science. 2009;324(5930):1064–1068.

7. Du Z, Aghoram K, Outlaw Jr WH. In VivoPhosphorylation of Phosphoenolpyruvate Carboxylase in Guard Cells ofVicia fabaL. Is Enhanced by Fusicoccin and Suppressed by Abscisic Acid. Archives of Biochemistry and Biophysics. 1997;337(2):345–350.

8. Bak G, Lee E-J, Lee Y, Kato M, Segami S, Sze H, et al. Rapid structural changes and acidification of guard cell vacuoles during stomatal closure require phosphatidylinositol 3, 5-bisphosphate. The Plant Cell. 2013;25(6):2202–2216.

9. Coursol S, Fan L-M, Le Stunff H, Spiegel S, Gilroy S, Assmann SM. Sphingolipid signalling in Arabidopsis guard cells involves heterotrimeric G proteins. Nature. 2003;423(6940):651–654.

10. Guo L, Mishra G, Markham JE, Li M, Tawfall A, Welti R, et al. Connections between sphingosine kinase and phospholipase D in the abscisic acid signaling pathway in Arabidopsis. J Biol Chem. 2012/01/26 ed. 2012 Mar;287(11):8286–96.

11. Worrall D, Liang YK, Alvarez S, Holroyd GH, Spiegel S, Panagopulos M, et al. Involvement of sphingosine kinase in plant cell signalling. Plant J. 2008/06/19 ed. 2008 Oct;56(1):64–72.

12. Guo L, Mishra G, Taylor K, Wang X. Phosphatidic acid binds and stimulates Arabidopsis sphingosine kinases. Journal of Biological Chemistry. 2011;286(15):13336–13345.

13. Lemichez E, Wu Y, Sanchez J-P, Mettouchi A, Mathur J, Chua N-H. Inactivation of AtRac1 by abscisic acid is essential for stomatal closure. Genes & development. 2001;15(14):1808–1816.

14. Dittrich P, Raschke K. Malate metabolism in isolated epidermis of Commelina communis L. in relation to stomatal functioning. Planta. 1977;134(1):77–81.

15. Hugouvieux V, Kwak JM, Schroeder JI. An mRNA cap binding protein, ABH1, modulates early abscisic acid signal transduction in Arabidopsis. Cell. 2001;106(4):477–487.

16. Geiger D, Maierhofer T, AL-Rasheid KA, Scherzer S, Mumm P, Liese A, et al. Stomatal closure by fast abscisic acid signaling is mediated by the guard cell anion channel SLAH3 and the receptor RCAR1. Science signaling. 2011;4(173):ra32–ra32.

17. Geiger D, Scherzer S, Mumm P, Stange A, Marten I, Bauer H, et al. Activity of guard cell anion channel SLAC1 is controlled by drought-stress signaling kinase-phosphatase pair. Proceedings of the National Academy of Sciences. 2009;106(50):21425–21430.

18. Umezawa T, Sugiyama N, Mizoguchi M, Hayashi S, Myouga F, Yamaguchi-Shinozaki K, et al. Type 2C protein phosphatases directly regulate abscisic acid-activated protein kinases in Arabidopsis. Proceedings of the National Academy of sciences. 2009;106(41):17588–17593.

19. Vlad F, Rubio S, Rodrigues A, Sirichandra C, Belin C, Robert N, et al. Protein phosphatases 2C regulate the activation of the Snf1-related kinase OST1 by abscisic acid in Arabidopsis. Plant Cell. 2009 Oct;21(10):3170–84.

20. Hua D, Wang C, He J, Liao H, Duan Y, Zhu Z, et al. A plasma membrane receptor kinase, GHR1, mediates abscisic acid-and hydrogen peroxide-regulated stomatal movement in Arabidopsis. The Plant Cell. 2012;24(6):2546–2561.

21. Geiger D, Scherzer S, Mumm P, Marten I, Ache P, Matschi S, et al. Guard cell anion channel SLAC1 is regulated by CDPK protein kinases with distinct Ca2+ affinities. Proc Natl Acad Sci U S A. 2010/04/14 ed. 2010 Apr 27;107(17):8023–8.

22. Zhang W, Fan L-M, Wu W-H. Osmo-sensitive and stretch-activated calcium-permeable channels in Vicia faba guard cells are regulated by actin dynamics. Plant Physiology. 2007;143(3):1140–1151.

23. Chakravorty D, Trusov Y, Zhang W, Acharya BR, Sheahan MB, McCurdy DW, et al. An atypical heterotrimeric G-protein gamma-subunit is involved in guard cell K(+)-channel regulation and morphological development in Arabidopsis thaliana. Plant J. 2011/05/18 ed. 2011 Sep;67(5):840–51.

24. Levchenko V, Konrad KR, Dietrich P, Roelfsema MR, Hedrich R. Cytosolic abscisic acid activates guard cell anion channels without preceding Ca2+ signals. Proc Natl Acad Sci U S A. 2005/03/09 ed. 2005 Mar 15;102(11):4203–8.

25. Hedrich R. Ion channels in plants. Physiological reviews. 2012;

26. Meyer S, Mumm P, Imes D, Endler A, Weder B, Al-Rasheid KA, et al. AtALMT12 represents an R-type anion channel required for stomatal movement in Arabidopsis guard cells. Plant J. 2010/07/16 ed. 2010 Sep;63(6):1054–62.

27. Jiang K, Sorefan K, Deeks MJ, Bevan MW, Hussey PJ, Hetherington AM. The ARP2/3 complex mediates guard cell actin reorganization and stomatal movement in Arabidopsis. The Plant Cell. 2012;24(5):2031–2040.

28. Szymanski DB. Breaking the WAVE complex: the point of Arabidopsis trichomes. Curr Opin Plant Biol. 2005 Feb;8(1):103–12.

29. Nagy SK, Darula Z, Kállai BM, Bögre L, Bánhegyi G, Medzihradszky KF, et al. Activation of AtMPK9 through autophosphorylation that makes it independent of the canonical MAPK cascades. Biochemical Journal. 2015;467(1):167–175.

30. Nakagawa N, Kato M, Takahashi Y, Shimazaki K, Tamura K, Tokuji Y, et al. Degradation of long-chain base 1-phosphate (LCBP) in Arabidopsis: functional characterization of LCBP phosphatase involved in the dehydration stress response. Journal of plant research. 2012;125(3):439–449.

31. Islam MM, Hossain MA, Jannat R, Munemasa S, Nakamura Y, Mori IC, et al. Cytosolic alkalization and cytosolic calcium oscillation in Arabidopsis guard cells response to ABA and MeJA. Plant and Cell Physiology. 2010;51(10):1721–1730.

32. Kim Y-M, Han Y-J, Hwang O-J, Lee S-S, Shin A-Y, Kim SY, et al. Overexpression of Arabidopsis translationally controlled tumor protein gene AtTCTP enhances drought tolerance with rapid ABA-induced stomatal closure. Molecules and cells. 2012;33(6):617–626.

33. Sanders D, Pelloux J, Brownlee C, Harper JF. Calcium at the crossroads of signaling. The Plant Cell. 2002;14(suppl 1):S401–S417.

34. Ward JM, Schroeder JI. Calcium-activated K+ channels and calcium-induced calcium release by slow vacuolar ion channels in guard cell vacuoles implicated in the control of stomatal closure. The Plant Cell. 1994;6(5):669–683.

35. Kinoshita T, Nishimura M, Shimazaki KI. Cytosolic concentration of Ca2+ regulates the plasma membrane H+-ATPase in guard cells of fava bean. The Plant Cell. 1995;7(8):1333–1342.

36. Pei ZM, Baizabal-Aguirre VM, Allen GJ, Schroeder JI. A transient outward-rectifying K+ channel current down-regulated by cytosolic Ca2+ in Arabidopsis thaliana guard cells. Proc Natl Acad Sci U S A. 1998 May 26;95(11):6548–53.

37. Scherzer S, Maierhofer T, Al-Rasheid KA, Geiger D, Hedrich R. Multiple calcium-dependent kinases modulate ABA-activated guard cell anion channels. Molecular Plant. 2012;5(6):1409–1412.

38. Pappan KL, Wang X. Assaying different types of plant phospholipase D activities in vitro. Methods Mol Biol. 2013;1009:205–17.

39. Otterhag L, Sommarin M, Pical C. N-terminal EF-hand-like domain is required for phosphoinositide-specific phospholipase C activity in Arabidopsis thaliana. FEBS letters. 2001;497(2–3):165–170.

40. Tang R-J, Liu H, Yang Y, Yang L, Gao X-S, Garcia VJ, et al. Tonoplast calcium sensors CBL2 and CBL3 control plant growth and ion homeostasis through regulating V-ATPase activity in Arabidopsis. Cell research. 2012;22(12):1650–1665.

41. Guse AH. Cyclic ADP-ribose: a novel Ca2+-mobilising second messenger. Cellular signalling. 1999;11(5):309–316.

42. Leckie CP, McAinsh MR, Allen GJ, Sanders D, Hetherington AM. Abscisic acid-induced stomatal closure mediated by cyclic ADP-ribose. Proc Natl Acad Sci U S A. 1998/12/23 ed. 1998 Dec 22;95(26):15837–42.

43. Schroeder JI, Hagiwara S. Repetitive increases in cytosolic Ca2+ of guard cells by abscisic acid activation of nonselective Ca2+ permeable channels. Proc Natl Acad Sci U S A. 1990 Dec;87(23):9305–9.

44. Gilroy S, Fricker MD, Read ND, Trewavas AJ. Role of Calcium in Signal Transduction of Commelina Guard Cells. Plant Cell. 1991/04/01 ed. 1991 Apr;3(4):333–44.

45. Grabov A, Blatt MR. Membrane voltage initiates Ca2+ waves and potentiates Ca2+ increases with abscisic acid in stomatal guard cells. Proceedings of the National Academy of Sciences. 1998;95(8):4778–4783.

46. Acharya BR, Jeon BW, Zhang W, Assmann SM. Open Stomata 1 (OST 1) is limiting in abscisic acid responses of Arabidopsis guard cells. New Phytologist. 2013;200(4):1049–1063.

47. Lemtiri-Chlieh F, MacRobbie EA, Webb AA, Manison NF, Brownlee C, Skepper JN, et al. Inositol hexakisphosphate mobilizes an endomembrane store of calcium in guard cells. Proceedings of the National Academy of Sciences. 2003;100(17):10091–10095.

48. Staxén I, Pical C, Montgomery LT, Gray JE, Hetherington AM, McAinsh MR. Abscisic acid induces oscillations in guard-cell cytosolic free calcium that involve phosphoinositide-specific phospholipase C. Proceedings of the National Academy of Sciences. 1999;96(4):1779–1784.

49. Mori IC, Murata Y, Yang Y, Munemasa S, Wang YF, Andreoli S, et al. CDPKs CPK6 and CPK3 function in ABA regulation of guard cell S-type anion- and Ca(2+)-permeable channels and stomatal closure. PLoS Biol. 2006/10/13 ed. 2006 Oct;4(10):e327.

50. Swatek KN, Wilson RS, Ahsan N, Tritz RL, Thelen JJ. Multisite phosphorylation of 14-3-3 proteins by calcium-dependent protein kinases. Biochemical Journal. 2014;459(1):15–25.

51. Brandt B, Brodsky DE, Xue S, Negi J, Iba K, Kangasjarvi J, et al. Reconstitution of abscisic acid activation of SLAC1 anion channel by CPK6 and OST1 kinases and branched ABI1 PP2C phosphatase action. Proc Natl Acad Sci U S A. 2012/06/13 ed. 2012 Jun 26;109(26):10593–8.

52. Munnik T, Irvine RF, Musgrave A. Phospholipid signalling in plants. Biochimica et Biophysica Acta (BBA)-Lipids and Lipid Metabolism. 1998;1389(3):222–272.

53. Hosy E, Vavasseur A, Mouline K, Dreyer I, Gaymard F, Porée F, et al. The Arabidopsis outward K+ channel GORK is involved in regulation of stomatal movements and plant transpiration. Proceedings of the National Academy of Sciences. 2003;100(9):5549–5554.

54. Zheng ZL, Nafisi M, Tam A, Li H, Crowell DN, Chary SN, et al. Plasma membrane-associated ROP10 small GTPase is a specific negative regulator of abscisic acid responses in Arabidopsis. Plant Cell. 2002 Nov;14(11):2787–97.

55. Allen GJ, Murata Y, Chu SP, Nafisi M, Schroeder JI. Hypersensitivity of abscisic acid-induced cytosolic calcium increases in the Arabidopsis farnesyltransferase mutant era1-2. Plant Cell. 2002/07/18 ed. 2002 Jul;14(7):1649–62.

56. Guo L, Devaiah SP, Narasimhan R, Pan X, Zhang Y, Zhang W, et al. Cytosolic glyceraldehyde-3-phosphate dehydrogenases interact with phospholipase Dδ to transduce hydrogen peroxide signals in the Arabidopsis response to stress. The Plant Cell. 2012;24(5):2200–2212.

57. Pandey S, Assmann SM. The Arabidopsis putative G protein–coupled receptor GCR1 interacts with the G protein α subunit GPA1 and regulates abscisic acid signaling. The Plant Cell. 2004;16(6):1616–1632.

58. Yu F, Qian L, Nibau C, Duan Q, Kita D, Levasseur K, et al. FERONIA receptor kinase pathway suppresses abscisic acid signaling in Arabidopsis by activating ABI2 phosphatase. Proceedings of the National Academy of Sciences. 2012;109(36):14693–14698.

59. Li Z, Liu D. ROPGEF1 and ROPGEF4 are functional regulators of ROP11 GTPase in ABA-mediated stomatal closure in Arabidopsis. FEBS letters. 2012;586(9):1253–1258.

60. Gookin TE, Assmann SM. Significant reduction of BiFC non-specific assembly facilitates in planta assessment of heterotrimeric G-protein interactors. Plant J. 2014/09/05 ed. 2014 Nov;80(3):553–67.

61. Zhao J, Wang X. Arabidopsis phospholipase Dalpha1 interacts with the heterotrimeric G-protein alpha-subunit through a motif analogous to the DRY motif in G-protein-coupled receptors. J Biol Chem. 2004 Jan 16;279(3):1794–800.

62. Mulaudzi T, Ludidi N, Ruzvidzo O, Morse M, Hendricks N, Iwuoha E, et al. Identification of a novel Arabidopsis thaliana nitric oxide-binding molecule with guanylate cyclase activity in vitro. FEBS letters. 2011;585(17):2693–2697.

63. Li S, Assmann SM, Albert R. Predicting essential components of signal transduction networks: a dynamic model of guard cell abscisic acid signaling. PLoS biology. 2006;4(10).

64. Merlot S, Leonhardt N, Fenzi F, Valon C, Costa M, Piette L, et al. Constitutive activation of a plasma membrane H(+)-ATPase prevents abscisic acid-mediated stomatal closure. EMBO J. 2007/06/09 ed. 2007 Jul 11;26(13):3216–26.

65. Boss WF, Im YJ. Phosphoinositide signaling. Annual review of plant biology. 2012;63:409–429.

66. Jiang Y, Wu K, Lin F, Qu Y, Liu X, Zhang Q. Phosphatidic acid integrates calcium signaling and microtubule dynamics into regulating ABA-induced stomatal closure in Arabidopsis. Planta. 2014;239(3):565–575.

67. Gardner MK, Zanic M, Howard J. Microtubule catastrophe and rescue. Current opinion in cell biology. 2013;25(1):14–22.

68. Hunt L, Lerner F, Ziegler M. NAD - new roles in signalling and gene regulation in plants. New Phytol. 2004 Jul;163(1):31–44.

69. Desikan R, Griffiths R, Hancock J, Neill S. A new role for an old enzyme: nitrate reductase-mediated nitric oxide generation is required for abscisic acid-induced stomatal closure in Arabidopsis thaliana. Proceedings of the National Academy of Sciences. 2002;99(25):16314–16318.

70. Sokolovski S, Blatt MR. Nitric oxide block of outward-rectifying K+ channels indicates direct control by protein nitrosylation in guard cells. Plant Physiology. 2004;136(4):4275–4284.

71. Sokolovski S, Hills A, Gay RA, Blatt MR. Functional interaction of the SNARE protein NtSyp121 in Ca2+ channel gating, Ca2+ transients and ABA signalling of stomatal guard cells. Mol Plant. 2008/03/01 ed. 2008 Mar;1(2):347–58.

72. Vahisalu T, Kollist H, Wang YF, Nishimura N, Chan WY, Valerio G, et al. SLAC1 is required for plant guard cell S-type anion channel function in stomatal signalling. Nature. 2008/02/29 ed. 2008 Mar 27;452(7186):487–91.

73. Imes D, Mumm P, Böhm J, Al-Rasheid KA, Marten I, Geiger D, et al. Open stomata 1 (OST 1) kinase controls R–type anion channel QUAC 1 in A rabidopsis guard cells. The Plant Journal. 2013;74(3):372–382.

74. Sasaki T, Mori IC, Furuichi T, Munemasa S, Toyooka K, Matsuoka K, et al. Closing plant stomata requires a homolog of an aluminum-activated malate transporter. Plant and cell physiology. 2010;51(3):354–365.

75. Sirichandra C, Gu D, Hu HC, Davanture M, Lee S, Djaoui M, et al. Phosphorylation of the Arabidopsis AtrbohF NADPH oxidase by OST1 protein kinase. FEBS Lett. 2009/09/01 ed. 2009 Sep 17;583(18):2982–6.

76. Ogasawara Y, Kaya H, Hiraoka G, Yumoto F, Kimura S, Kadota Y, et al. Synergistic activation of the Arabidopsis NADPH oxidase AtrbohD by Ca2+ and phosphorylation. J Biol Chem. 2008/01/26 ed. 2008 Apr 4;283(14):8885–92.

77. Kimura S, Kaya H, Kawarazaki T, Hiraoka G, Senzaki E, Michikawa M, et al. Protein phosphorylation is a prerequisite for the Ca2+-dependent activation of Arabidopsis NADPH oxidases and may function as a trigger for the positive feedback regulation of Ca2+ and reactive oxygen species. Biochim Biophys Acta. 2011/10/18 ed. 2012 Feb;1823(2):398–405.

78. Grondin A, Rodrigues O, Verdoucq L, Merlot S, Leonhardt N, Maurel C. Aquaporins contribute to ABA-triggered stomatal closure through OST1-mediated phosphorylation. The Plant Cell. 2015;27(7):1945–1954.

79. Mishra G, Zhang W, Deng F, Zhao J, Wang X. A bifurcating pathway directs abscisic acid effects on stomatal closure and opening in Arabidopsis. Science. 2006;312(5771):264–266.

80. Zhang W, Qin C, Zhao J, Wang X. Phospholipase Dα1-derived phosphatidic acid interacts with ABI1 phosphatase 2C and regulates abscisic acid signaling. Proceedings of the National Academy of Sciences. 2004;101(25):9508–9513.

81. Zhang Y, Zhu H, Zhang Q, Li M, Yan M, Wang R, et al. Phospholipase Dα1 and phosphatidic acid regulate NADPH oxidase activity and production of reactive oxygen species in ABA-mediated stomatal closure in Arabidopsis. The Plant Cell. 2009;21(8):2357–2377.

82. Miedema H, Assmann SM. A membrane-delimited effect of internal pH on the K+ outward rectifier of Vicia faba guard cells. The Journal of membrane biology. 1996;154(3):227–237.

83. Luo H, Morsomme P, Boutry M. The two major types of plant plasma membrane H+-ATPases show different enzymatic properties and confer differential pH sensitivity of yeast growth. Plant physiology. 1999;119(2):627–634.

84. Leube MP, Grill E, Amrhein N. ABI1 of Arabidopsis is a protein serine/threonine phosphatase highly regulated by the proton and magnesium ion concentration 1. FEBS letters. 1998;424(1–2):100–104.

85. Puli MR, Rajsheel P, Aswani V, Agurla S, Kuchitsu K, Raghavendra AS. Stomatal closure induced by phytosphingosine-1-phosphate and sphingosine-1-phosphate depends on nitric oxide and pH of guard cells in Pisum sativum. Planta. 2016;244(4):831–841.

86. Suhita D, Raghavendra AS, Kwak JM, Vavasseur A. Cytoplasmic alkalization precedes reactive oxygen species production during methyl jasmonate-and abscisic acid-induced stomatal closure. Plant physiology. 2004;134(4):1536–1545.

87. Jung J-Y, Kim Y-W, Kwak JM, Hwang J-U, Young J, Schroeder JI, et al. Phosphatidylinositol 3-and 4-phosphate are required for normal stomatal movements. The Plant Cell. 2002;14(10):2399–2412.

88. Uraji M, Katagiri T, Okuma E, Ye W, Hossain MA, Masuda C, et al. Cooperative function of PLDdelta and PLDalpha1 in abscisic acid-induced stomatal closure in Arabidopsis. Plant Physiol. 2012/03/07 ed. 2012 May;159(1):450–60.

89. Lee SC, Lan W, Buchanan BB, Luan S. A protein kinase-phosphatase pair interacts with an ion channel to regulate ABA signaling in plant guard cells. Proc Natl Acad Sci U S A. 2009/12/04 ed. 2009 Dec 15;106(50):21419–24.

90. Choi Y, Lee Y, Jeon BW, Staiger CJ, Lee Y. Phosphatidylinositol 3- and 4-phosphate modulate actin filament reorganization in guard cells of day flower. Plant Cell Environ. 2007/12/20 ed. 2008 Mar;31(3):366–77.

91. Kwak JM, Moon J-H, Murata Y, Kuchitsu K, Leonhardt N, DeLong A, et al. Disruption of a guard cell–expressed protein phosphatase 2A regulatory subunit, RCN1, confers abscisic acid insensitivity in Arabidopsis. The Plant Cell. 2002;14(11):2849–2861.

92. Antoni R, Gonzalez-Guzman M, Rodriguez L, Rodrigues A, Pizzio GA, Rodriguez PL. Selective inhibition of clade A phosphatases type 2C by PYR/PYL/RCAR abscisic acid receptors. Plant physiology. 2012;158(2):970–980.

93. Li Z, Li Z, Gao X, Chinnusamy V, Bressan R, Wang Z-X, et al. ROP11 GTPase negatively regulates ABA signaling by protecting ABI1 phosphatase activity from inhibition by the ABA receptor RCAR1/PYL9 in Arabidopsis. Journal of Integrative Plant Biology. 2012;54(3):180–188.

94. Li Z, Kang J, Sui N, Liu D. ROP11 GTPase is a negative regulator of multiple ABA responses in Arabidopsis. Journal of integrative plant biology. 2012;54(3):169–179.

95. Köhler B, Hills A, Blatt MR. Control of guard cell ion channels by hydrogen peroxide and abscisic acid indicates their action through alternate signaling pathways. Plant physiology. 2003;131(2):385–388.

96. Zhang X, Wang H, Takemiya A, Song C, Kinoshita T, Shimazaki K. Inhibition of blue light-dependent H+ pumping by abscisic acid through hydrogen peroxide-induced dephosphorylation of the plasma membrane H+-ATPase in guard cell protoplasts. Plant Physiology. 2004;136(4):4150–4158.

97. Meinhard M, Grill E. Hydrogen peroxide is a regulator of ABI1, a protein phosphatase 2C from Arabidopsis. Febs Letters. 2001;508(3):443–446.

98. Sridharamurthy M, Kovach A, Zhao Y, Zhu JK, Xu HE, Swaminathan K, et al. H2O2 inhibits ABA-signaling protein phosphatase HAB1. PLoS One. 2014;9(12):e113643.

99. Meinhard M, Rodriguez PL, Grill E. The sensitivity of ABI2 to hydrogen peroxide links the abscisic acid-response regulator to redox signalling. Planta. 2002;214(5):775–782.

100. Zhao Y, Zhao S, Mao T, Qu X, Cao W, Zhang L, et al. The plant-specific actin binding protein SCAB1 stabilizes actin filaments and regulates stomatal movement in Arabidopsis. The Plant Cell. 2011;23(6):2314–2330.

101. Guo L, Wang X. Crosstalk between Phospholipase D and Sphingosine Kinase in Plant Stress Signaling. Frontiers in plant science. 2012;3:51–51.

102. Gary JD, Wurmser AE, Bonangelino CJ, Weisman LS, Emr SD. Fab1p is essential for PtdIns(3)P 5-kinase activity and the maintenance of vacuolar size and membrane homeostasis. J Cell Biol. 1998/10/08 ed. 1998 Oct 5;143(1):65–79.

103. Baars TL, Petri S, Peters C, Mayer A. Role of the V-ATPase in regulation of the vacuolar fission-fusion equilibrium. Mol Biol Cell. 2007/07/27 ed. 2007 Oct;18(10):3873–82.

104. Pei ZM, Murata Y, Benning G, Thomine S, Klusener B, Allen GJ, et al. Calcium channels activated by hydrogen peroxide mediate abscisic acid signalling in guard cells. Nature. 2000 Aug 17;406(6797):731–4.

105. Gonugunta VK, Srivastava N, Puli MR, Raghavendra AS. Nitric oxide production occurs after cytosolic alkalinization during stomatal closure induced by abscisic acid. Plant, Cell & Environment. 2008;31(11):1717–1724.

106. Bright J, Desikan R, Hancock JT, Weir IS, Neill SJ. ABA-induced NO generation and stomatal closure in Arabidopsis are dependent on H2O2 synthesis. The Plant Journal. 2006;45(1):113–122.

107. MacRobbie EA, Kurup S. Signalling mechanisms in the regulation of vacuolar ion release in guard cells. New Phytol. 2007/08/11 ed. 2007;175(4):630–40.

108. MacRobbie EA. ABA activates multiple Ca(2+) fluxes in stomatal guard cells, triggering vacuolar K(+)(Rb(+)) release. Proc Natl Acad Sci U S A. 2000/10/12 ed. 2000 Oct 24;97(22):12361–8.

109. Eisinger W, Ehrhardt D, Briggs W. Microtubules are essential for guard-cell function in Vicia and Arabidopsis. Molecular Plant. 2012;5(3):601–610.

110. Gilroy S, Read N, Trewavas AJ. Elevation of cytoplasmic calcium by caged calcium or caged inositol trisphosphate initiates stomatal closure. Nature. 1990;346(6286):769–771.

111. Chen ZH, Hills A, Lim CK, Blatt MR. Dynamic regulation of guard cell anion channels by cytosolic free Ca2+ concentration and protein phosphorylation. Plant J. 2009/12/18 ed. 2010 Mar;61(5):816–25.

112. Siegel RS, Xue S, Murata Y, Yang Y, Nishimura N, Wang A, et al. Calcium elevation-dependent and attenuated resting calcium-dependent abscisic acid induction of stomatal closure and abscisic acid-induced enhancement of calcium sensitivities of S-type anion and inward-rectifying K channels in Arabidopsis guard cells. Plant J. 2009/03/24 ed. 2009 Jul;59(2):207–20.

113. Distefano AM, GARCÍA-MATA C, Lamattina L, Laxalt AM. Nitric oxide-induced phosphatidic acid accumulation: a role for phospholipases C and D in stomatal closure. Plant, cell & environment. 2008;31(2):187–194.

114. Dubovskaya LV, Bakakina YS, Kolesneva EV, Sodel DL, McAinsh MR, Hetherington AM, et al. cGMP-dependent ABA-induced stomatal closure in the ABA-insensitive Arabidopsis mutant abi1-1. New Phytol. 2011/03/05 ed. 2011 Jul;191(1):57–69.

115. Kuhn JM, Boisson-Dernier A, Dizon MB, Maktabi MH, Schroeder JI. The protein phosphatase AtPP2CA negatively regulates abscisic acid signal transduction in Arabidopsis, and effects of abh1 on AtPP2CA mRNA. Plant physiology. 2006;140(1):127–139.

116. Zhang X, Zhang L, Dong F, Gao J, Galbraith DW, Song C-P. Hydrogen peroxide is involved in abscisic acid-induced stomatal closure in Vicia faba. Plant physiology. 2001;126(4):1438–1448.

117. Zhang W, Jeon BW, Assmann SM. Heterotrimeric G-protein regulation of ROS signalling and calcium currents in Arabidopsis guard cells. Journal of Experimental Botany. 2011;62(7):2371–2379.

118. Ng CK-Y, Carr K, McAinsh MR, Powell B, Hetherington AM. Drought-induced guard cell signal transduction involves sphingosine-1-phosphate. Nature. 2001;410(6828):596–599.

119. Murata Y, Pei Z-M, Mori IC, Schroeder J. Abscisic acid activation of plasma membrane Ca2+ channels in guard cells requires cytosolic NAD (P) H and is differentially disrupted upstream and downstream of reactive oxygen species production in abi1-1 and abi2-1 protein phosphatase 2C mutants. The Plant Cell. 2001;13(11):2513–2523.

120. Macrobbie EA. Signalling in guard cells and regulation of ion channel activity. J Exp Bot. 1997 Mar;48 Spec No:515–28.

121. Meimoun P, Vidal G, Bohrer AS, Lehner A, Tran D, Briand J, et al. Intracellular Ca2+ stores could participate to abscisic acid-induced depolarization and stomatal closure in Arabidopsis thaliana. Plant Signal Behav. 2009/10/23 ed. 2009 Sep;4(9):830–5.

122. Jammes F, Song C, Shin D, Munemasa S, Takeda K, Gu D, et al. MAP kinases MPK9 and MPK12 are preferentially expressed in guard cells and positively regulate ROS-mediated ABA signaling. Proceedings of the National Academy of sciences. 2009;106(48):20520–20525.

123. Suh SJ, Wang Y-F, Frelet A, Leonhardt N, Klein M, Forestier C, et al. The ATP binding cassette transporter AtMRP5 modulates anion and calcium channel activities in Arabidopsis guard cells. Journal of Biological Chemistry. 2007;282(3):1916–1924.

124. Wang X-Q, Ullah H, Jones AM, Assmann SM. G protein regulation of ion channels and abscisic acid signaling in Arabidopsis guard cells. Science. 2001;292(5524):2070–2072.

125. Distefano AM, Scuffi D, Garcia-Mata C, Lamattina L, Laxalt AM. Phospholipase Ddelta is involved in nitric oxide-induced stomatal closure. Planta. 2012/08/31 ed. 2012 Dec;236(6):1899–907.

126. Park KY, Jung JY, Park J, Hwang JU, Kim YW, Hwang I, et al. A role for phosphatidylinositol 3-phosphate in abscisic acid-induced reactive oxygen species generation in guard cells. Plant Physiol. 2003/05/15 ed. 2003 May;132(1):92–8.

127. Ellson CD, Gobert-Gosse S, Anderson KE, Davidson K, Erdjument-Bromage H, Tempst P, et al. PtdIns(3)P regulates the neutrophil oxidase complex by binding to the PX domain of p40(phox). Nat Cell Biol. 2001/07/04 ed. 2001 Jul;3(7):679–82.

128. Saito N, Munemasa S, Nakamura Y, Shimoishi Y, Mori IC, Murata Y. Roles of RCN1, regulatory A subunit of protein phosphatase 2A, in methyl jasmonate signaling and signal crosstalk between methyl jasmonate and abscisic acid. Plant Cell Physiol. 2008/07/25 ed. 2008 Sep;49(9):1396–401.

129. Negi J, Matsuda O, Nagasawa T, Oba Y, Takahashi H, Kawai-Yamada M, et al. CO 2 regulator SLAC1 and its homologues are essential for anion homeostasis in plant cells. Nature. 2008;452(7186):483–486.

130. Brandt B, Munemasa S, Wang C, Nguyen D, Yong T, Yang PG, et al. Calcium specificity signaling mechanisms in abscisic acid signal transduction in Arabidopsis guard cells. Elife. 2015;4:e03599.

131. Maheshwari P, Assmann SM, Albert R. A guard cell abscisic acid (ABA) network model that captures the stomatal resting state. Frontiers in Physiology. 2020;11:927.

132. Martinoia E, Maeshima M, Neuhaus HE. Vacuolar transporters and their essential role in plant metabolism. Journal of experimental botany. 2007;58(1):83–102.

133. Ferjani A, Segami S, Horiguchi G, Muto Y, Maeshima M, Tsukaya H. Keep an eye on PPi: the vacuolar-type H+-pyrophosphatase regulates postgerminative development in Arabidopsis. The Plant Cell. 2011;23(8):2895–2908.

134. Krebs M, Beyhl D, Görlich E, Al-Rasheid KA, Marten I, Stierhof Y-D, et al. Arabidopsis V-ATPase activity at the tonoplast is required for efficient nutrient storage but not for sodium accumulation. Proceedings of the National Academy of Sciences. 2010;107(7):3251–3256.

135. Schwartz A. Role of Ca2+ and EGTA on Stomatal Movements in Commelina communis L. Plant Physiology. 1985;79(4):1003–1005.

136. Merlot S, Gosti F, Guerrier D, Vavasseur A, Giraudat J. The ABI1 and ABI2 protein phosphatases 2C act in a negative feedback regulatory loop of the abscisic acid signalling pathway. The Plant Journal. 2001;25(3):295–303.

137. Saez A, Robert N, Maktabi MH, Schroeder JI, Serrano R, Rodriguez PL. Enhancement of abscisic acid sensitivity and reduction of water consumption in Arabidopsis by combined inactivation of the protein phosphatases type 2C ABI1 and HAB1. Plant physiology. 2006;141(4):1389–1399.

138. Rubio S, Rodrigues A, Saez A, Dizon MB, Galle A, Kim T-H, et al. Triple loss of function of protein phosphatases type 2C leads to partial constitutive response to endogenous abscisic acid. Plant physiology. 2009;150(3):1345–1355.
